# Astrocytic PCBP1 Suppresses Ferroptosis to Restore Glutamatergic Homeostasis and Mitigate Stress‐Induced Depression in Male Mice

**DOI:** 10.1002/advs.202513438

**Published:** 2025-12-12

**Authors:** Jinyu Zhang, Binbin Zhao, Min Jia, Yan Zhao, Ye Lu, Wenyu Xi, Ziyu Zhu, Xiaojuan Gong, Qingyan Ma, Yuan Gao, Yijie Guo, Pan Li, Feng Zhu, Shuguang Wei, Xiancang Ma, Yunpeng Wang

**Affiliations:** ^1^ Department of Psychiatry Shaanxi Key Laboratory of Biological Psychiatry First Affiliated Hospital of Xi'an Jiaotong University Xi'an Shaanxi 710061 China; ^2^ College of Forensic Science Xi'an Jiaotong University Xi'an Shaanxi 710061 China; ^3^ Department of Basic Medicine Xi'an Medical University Xi'an Shaanxi 710021 China; ^4^ Department of Computer Science City University of Hong Kong Kowloon Hong Kong 999077 China

**Keywords:** astrocyte, depression, ferroptosis, glutamate toxicity, PCBP1, ventral hippocampus

## Abstract

Major depressive disorder (MDD) is a critical psychiatric illness with significant societal implications. The exact molecular mechanisms underlying MDD, specifically concerning cellular iron metabolism and ferroptosis, remain inadequately characterized. This study explores the regulatory function of astrocytic ferroptosis and its linkage to depressive‐like behaviors in mice under chronic unpredictable mild stress. Through integrated proteomic and phosphoproteomic analyses, it is identified that the iron‐chaperone protein polyC‐RNA‐binding protein 1 (PCBP1) is a critical regulator of ferroptosis in astrocytes within the ventral hippocampus (vHip), which is closely linked to depressive‐like behaviors in mice. The reduction in PCBP1 in astrocytes heightens their vulnerability to ferroptosis, resulting in depressive‐like behaviors under subthreshold stress conditions. Conversely, pharmacological inhibition of ferroptosis or overexpression of PCBP1 in astrocytes counteracts stress‐induced depressive‐like behaviors, indicating a protective function for PCBP1. Mechanistically, PCBP1‐mediated astrocytic ferroptosis compromises glutamate clearance and disrupts glutamatergic neuronal activity. Significantly, astrocyte‐specific overexpression of PCBP1 in vHip mitigates chronic stress‐induced glutamate toxicity and restores neuronal function, leading to improvements in depressive‐like behaviors. These results highlight the crucial role of PCBP1 in astrocytic ferroptosis and emphasize its potential as a therapeutic target for MDD, providing novel perspectives on the pathophysiology of stress‐induced depression.

## Graphical abstract

This study demonstrates that polyC‐RNA‐binding protein 1 (PCBP1) in ventral hippocampal astrocytes modulates depressive‐like behaviors by regulating glutathione peroxidase 4‐mediated ferroptosis and synaptic glutamatergic transmission. PCBP1 overexpression intervention in the chronic unpredictable mild stress model rescues behavioral deficits, normalizes astrocytic PCBP1 expression, and restores neuronal excitability and mEPSC properties, highlighting a critical astrocyte‐neuron interaction in depression pathophysiology.

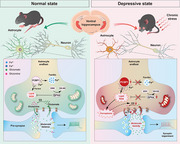

## 1 Introduction

Stress‐related neuropsychiatric disorders, particularly major depressive disorder (MDD), affect millions of individuals worldwide and contribute significantly to the rising rates of suicide.^[^ ^1^, ^2^ ^]^ Depressive symptoms can be persistent and recurrent, severely affecting quality of life and exacerbating the economic burden on families and society.^[^ ^3^ ^]^ Although current treatments, such as antidepressants and psychotherapy, provide relief for some individuals, a significant proportion of patients remain resistant to these interventions.^[^ ^4^ ^]^ Elucidating the pathophysiological mechanisms of stress‐related MDD is crucial for advancing the development of effective treatment strategies.

The hippocampus exhibits functional differentiation along its longitudinal axis, with the ventral hippocampus (vHip) involved in mood and stress regulation, while the dorsal hippocampus (dHip) is involved in learning and memory.^[^ ^5^ ^]^ Stress‐induced depressive‐like behaviors are more closely associated with neurobiological changes in the vHip, indicating its greater vulnerability.^[^ ^6^ ^]^ Restoration of vHip function is also a key mechanism underlying antidepressant efficacy.^[^ ^7^ ^]^ Astrocytes are essential for maintaining neuronal function by regulating neurotransmitter levels, supporting synaptic plasticity, and providing neuroprotection. Astrocytes help mitigate the neurotoxic effects of glutamate accumulation caused by chronic stress.^[^ ^8^ ^]^ Recent investigations involving both human patients with MDD and animal models have suggested that the reduction and dysfunction of astrocytes in the hippocampal region are involved in the pathogenesis of depression.^[^ ^9^, ^10^ ^]^ However, the mechanisms underlying the loss of astrocytes in depression remain unclear.

Ferroptosis is a newly recognized form of programmed cell death, characterized by iron‐dependent accumulation of lipid peroxides and reduced glutathione (GSH) synthesis.^[^ ^11^ ^]^ Recent studies highlight the crucial role of ferroptosis in various neurological disorders, such as Alzheimer's disease and Parkinson's disease.^[^ ^12^, ^13^ ^]^ Importantly, dysregulated iron metabolism and ferroptosis activation in the hippocampus contribute significantly to the pathophysiology of depression.^[^ ^14^ ^]^ Iron overload causes synaptic damage to hippocampal neurons in mice, while treatment with the iron chelator deferoxamine mesylate (DFO) alleviates depressive‐like behaviors in mice induced by chronic stress.^[^ ^15^ ^]^ PolyC‐RNA‐binding protein 1 (PCBP1), a critical iron chaperone, maintains intracellular iron homeostasis by facilitating iron delivery to ferritin.^[^ ^16^ ^]^ Abnormal expression of PCBP1 disrupts iron storage, promotes iron accumulation and lipid peroxidation, and sensitizes cells to ferroptosis.^[^ ^17^ ^]^ Emerging evidence highlights its pivotal role in the pathogenesis of neurodegenerative disorders.^[^ ^18^, ^19^, ^20^ ^]^ This evidence sheds light on the possibility that ferroptosis pathways may be a new therapeutic target for depression and warrant further investigation.

In this study, we employed the chronic unpredictable mild stress (CUMS) paradigm to investigate the pathophysiological mechanisms of depression in mice. The proteomic and phosphoproteomic analyses of the vHip suggested a potential involvement of the ferroptosis signaling pathway. Subsequent experiments demonstrated that PCBP1‐mediated ferroptosis in astrocytes disrupts both intercellular glutamate clearance and glutamatergic neuronal activity. Astrocyte‐specific overexpression of PCBP1 ameliorated chronic stress‐induced glutamate toxicity and restored glutamatergic neuronal activity, leading to an improvement in depressive‐like behaviors. This research highlights the crucial role of PCBP1 in astrocytic ferroptosis, offering a new basis for therapies for stress‐induced depression.

## 2 Results

### 2.1 Chronic Stress Affects Ferroptosis in vHip

**Figure 1:**
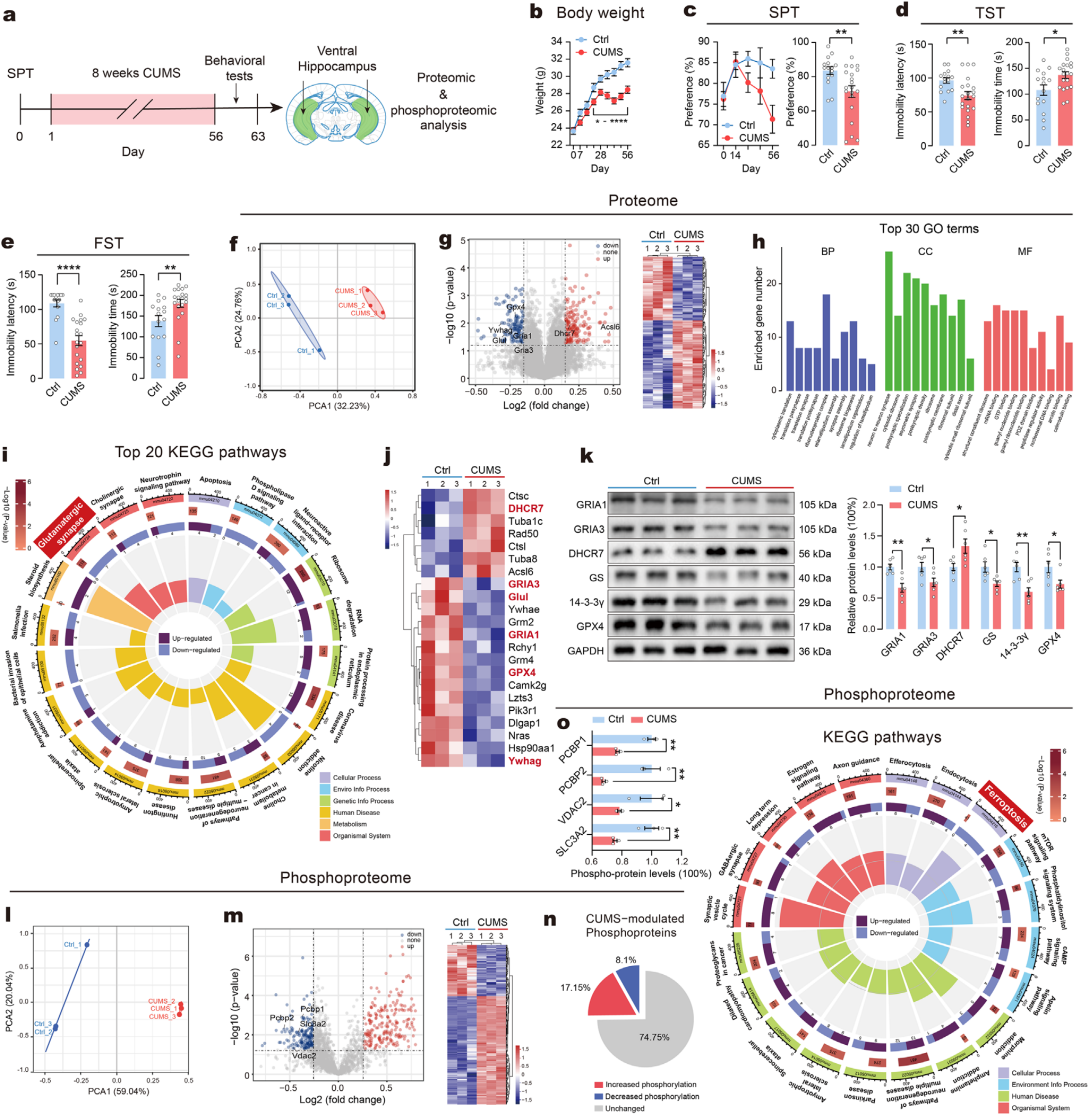
Chronic stress affects ferroptosis in vHip. a) Timeline of the CUMS paradigm and behavioral assessments in mice, followed by vHip tissue proteomic and phosphoproteomic sequencing. b) Body weight of mice during 8 weeks of CUMS. c) Sucrose preference of mice during 8 weeks of CUMS. d) Immobility latency and immobility time in TST. e) Immobility latency and immobility time in FST. *n* = 15–20 mice per group. f) Principal component analysis (PCA) results for proteomics obtained for vHip. g) Volcano map (left) and heatmap (right) of proteomics’ DEPs in CUMS mice. Labeled proteins represent key glutamatergic synapse‐ and ferroptosis‐associated molecules. h) Top 10 enriched biological process (BP), cellular component (CC), and molecular function (MF) terms in GO enrichment analysis of DEPs. i) Top 20 enriched KEGG pathways of DEPs. j) Heatmap of proteomics’ DEPs related to glutamatergic synapse and ferroptosis pathway. Red‐labeled proteins were validated by Western blot. k) Left: Representative immunoblots. Right: Expression levels of the DEPs validated by immunoblots. *n* = 6 samples/group for analysis. l) PCA results for phosphoproteomics obtained for vHip. m) Volcano map (left) and heatmap (right) of phosphoproteomics’ DEPs. Labeled proteins represent key ferroptosis‐associated molecules. n) Distribution of phosphoproteins modulated by CUMS. o) Left: Expression levels of phosphoproteins related to ferroptosis. Right: Enriched KEGG pathways of phosphoproteomics’ DEPs. Statistical analyses included unpaired t‐test, Welch's test, Mann‐Whitney test and two‐way ANOVA followed by Sidak's post hoc test. Data are presented as means ± SEM. CUMS versus Ctrl, *P < 0.05, **P < 0.01, ****P < 0.0001.

To investigate the effects of chronic physical and psychological stress on vHip cells, we used the widely recognized CUMS paradigm and subsequently assessed depressive‐like behaviors in mice (**Figure** **1**a). We evaluated the body weight of mice weekly during the CUMS period and found a significant weight loss in the CUMS mice compared to the non‐stressed control (Ctrl) mice from week 3 to week 8 (Figure 1b). Anhedonia and behavioral despair, the core symptoms of depressive phenotypes in mice, were determined by sucrose preference test (SPT), tail suspension test (TST) and forced swim test (FST) at the end of the CUMS. The sucrose preference began to show a continuous decrease from week 4 after CUMS and reached significance in week 8 (day 56) (Figure 1c). In addition, CUMS mice exhibited significantly decreased immobility latency and increased immobility time in both TST and FST (Figure 1d,e). These results suggested that the CUMS paradigm successfully induced depressive phenotypes in mice. Additionally, results of open field test (OFT) showed no significant differences in locomotor activity between groups (Figure S1a, Supporting Information), indicating that the observed behavioral alterations were not due to general motor impairments.

Next, we performed a comparative analysis of proteomic and phosphoproteomic expressions in vHip tissue from CUMS and Ctrl mice (*n* = 3/per group, each sample was pooled from three mice). In total, 7190 proteins and 5667 phosphorylation sites mapping to 2012 proteins were identified with a false discovery rate (FDR) below 1%. For the proteomics, principal component analysis (PCA) clearly classified vHip samples by group (Figure 1f), while Pearson correlation coefficients for biological replicates exceeding 0.99 (Figure S1b, Supporting Information), demonstrating highly accurate and reproducible results. A total of 271 differentially expressed proteins (DEPs) were identified in the CUMS group compared to the Ctrl group, including 157 up‐regulated proteins (2.18%) and 114 down‐regulated proteins (1.59%) (Figure S1c, Supporting Information also see Table S1A, Supporting Information). The volcano plot and heatmap demonstrated unique alterations in DEPs in the CUMS group, highlighting the dysregulated protein expression in the vHip (Figure 1g). Gene Ontology (GO) enrichment analysis of DEPs revealed the top 30 terms, with most biological processes and cellular components related to synaptic structure or functions and ribosome activity (Figure 1h; also see Table S2A, Supporting Information). The identified molecular functions predominantly involved small molecule, mRNA, and nucleotide binding, as well as peptidase regulator activity. Further Kyoto Encyclopedia of Genes and Genomes (KEGG) pathway enrichment analysis indicated that DEPs were involved primarily in pathways such as “ribosome”, “RNA degradation”, “glutamatergic synapse”, “pathways of neurodegeneration”, “nicotine addiction”, “apoptosis”, etc. (Figure 1i; also see Table S2B, Supporting Information). In particular, the “glutamatergic synapse” (mmu04724), known to be correlated with depression,^[^ ^21^ ^]^ was one of the most significantly enriched pathways in the KEGG category of the “organismal system” (enrichment factor 0.6087, *P* = 0.00169). Interestingly, we also noticed that DEPs were enriched in pathways related to cell death, including those related to ferroptosis‐associated proteins such as GPX4, ACSL6, and DHCR7 (Figure 1j). Emerging studies have increasingly recognized ferroptosis as a potential factor in the onset and progression of depression.^[^ ^22^, ^23^, ^24^ ^]^ We validated the expression levels of some key DEPs related to glutamatergic synapse and ferroptosis by immunoblotting. Notably, GRIA1, GRIA3, glutamine synthetase (GS), 14‐3‐3γ and GPX4 were significantly downregulated, while DHCR7 was significantly upregulated in CUMS group (Figure 1k).

The vHip phosphoproteomic analysis demonstrated an effective grouping (Figure 1l) and identified 345 upregulated phosphoproteins (17.15%) and 163 downregulated phosphoproteins (8.1%) in the CUMS group compared to the Ctrl group (Figure 1m,n; also see Table S1B, Supporting Information). KEGG enrichment analysis revealed that the main enriched pathways such as “efferocytosis”, “endocytosis”, “ferroptosis”, “morphine addiction”, “synaptic vesicle cycle”, etc. (Figure 1o; also see Table S2C, Supporting Information). We noticed that phosphoproteomic data also highlighted dysregulation of the ferroptosis pathway (including downregulated phosphorylation levels of the ferroptosis‐associated proteins PCBP1, PCBP2, VDAC2, and SLC3A2), consistent with the proteomic findings. The decreased expression of phospho‐PCBP1 and phospho‐PCBP2 in the CUMS group were validated by immunoblotting (Figure S1e,f, Supporting Information). Collectively, these results suggest that CUMS induced proteomic and phosphoproteomic signatures in vHip of mice, potentially involving ferroptosis‐related pathways. Therefore, we further investigated the mechanisms by which ferroptosis might affect vHip function and depressive‐like behaviors.

### 2.2 Chronic Stress Induces Astrocyte Ferroptosis and Neuronal Impairment in vHip

**Figure 2:**
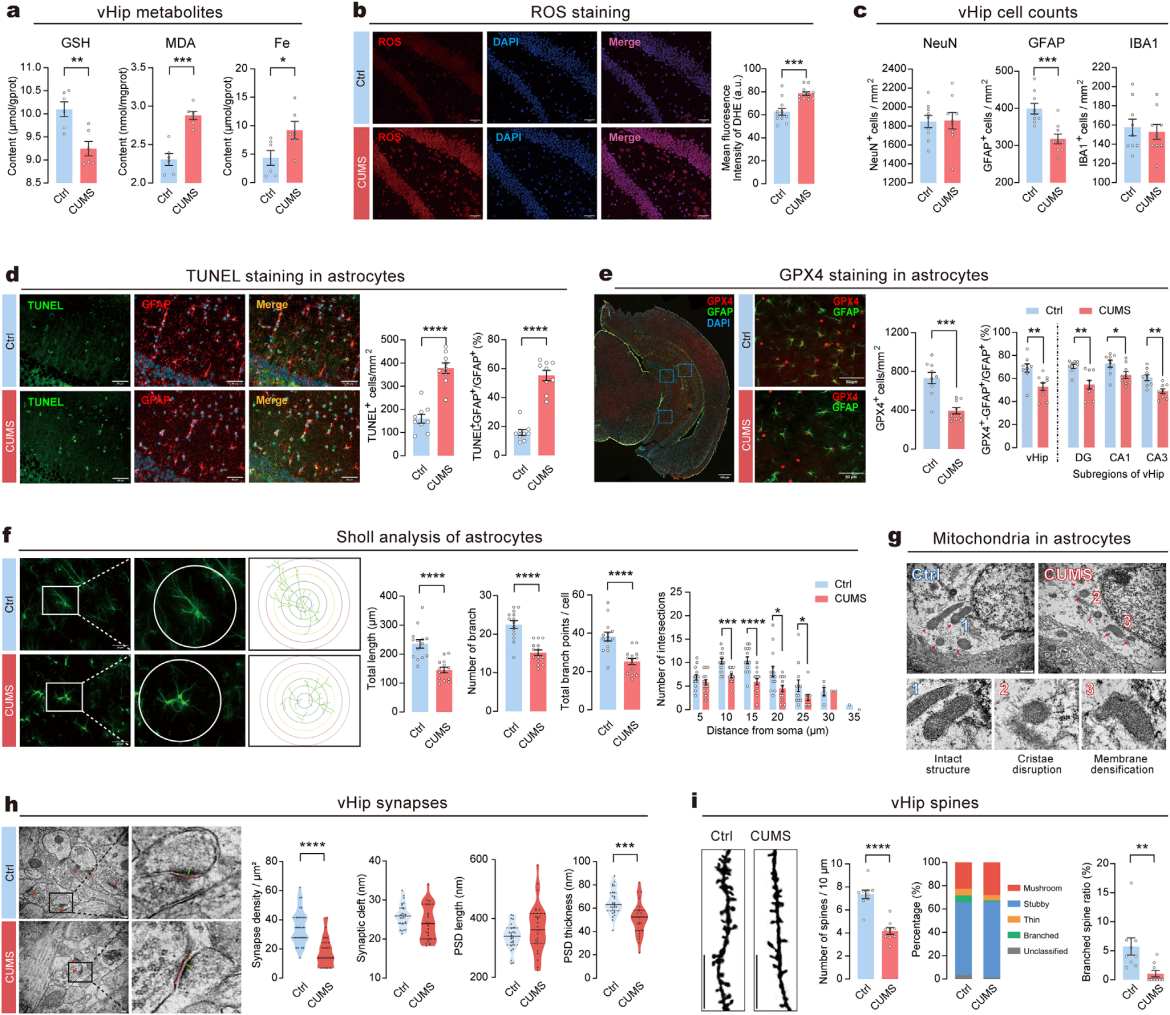
Chronic stress induces astrocyte ferroptosis and neuronal impairment in vHip. a) Levels of GSH, MDA, and iron in vHip. *n* = 6 samples per group. b) Left: Representative fluorescent images of ROS staining in vHip. Right: Mean fluorescence intensity of DHE in vHip. Scale bar = 40 µm. *n* = 12 slices from 3 animals per group. c) Quantification of neurons, astrocytes, and microglia cell numbers in the vHip between CUMS and Ctrl group. *n* = 9 slices from 3 animals/group. d) Apoptosis detection in vHip astrocytes by TUNEL staining (green). Scale bar = 50 µm. *n* = 9 slices from 3 animals/group. e) GPX4 expression in astrocytes of vHip. Left: Representative immunofluorescence images (red, GPX4; green, GFAP) at low‐magnification (Scale bar = 500 µm) and high‐magnification (Scale bar = 50 µm). Middle: Quantification of the GPX4‐positive cells in vHip. Right: Quantification of GPX4‐positive astrocytes in vHip and subregions (DG, CA1, and CA3). *n* = 9 slices from 3 animals per group. f) Sholl analysis of astrocytes in vHip. Left: Representative images of astrocytic morphology. Right: Quantitative analysis of total branch length, number of branches, and branch points per cell. Scale bar = 20 µm. *n* = 14 cells pergroup. g) Representative transmission electron microscopy (TEM) images of mitochondrial morphology in vHip astrocytes. Mitochondria exhibited intact morphology and structure in Ctrl group (subpanel 1). The mitochondrial structure was unclear, with increased double membrane density and disrupted cristae in CUMS group (subpanel 2 and 3). Scale bar = 500 nm. h) Ultrastructural analysis of synapses in vHip neurons. Left: Representative TEM images showing the ultrastructure of vHip neurons. Right: Quantitative analysis of synaptic ultrastructure in vHip neurons. Scale bar = 200 nm. i) Analysis of dendritic spine morphology in vHip. Left: Representative Golgi‐Cox staining images. Right: Quantification of spine density and distribution of spine types. Scale bars = 10 µm. Statistical analyses included unpaired t‐test, Welch's test and Mann‐Whitney test. Data are presented as means ± SEM. *P < 0.05, **P < 0.01, ***P < 0.001, ****P < 0.0001.

To confirm whether the ferroptosis pathway in vHip is associated with chronic stress‐induced depressive‐like behaviors, we measured several pathological phenomena associated with ferroptosis in vHip. Chronic stress significantly reduced the level of GSH in vHip while significantly increasing MDA (a marker of lipid peroxidation) and Fe content (**Figure** **2**a). Using dihydroethidium staining, we evaluated ROS levels, a hallmark of ferroptosis, and found exacerbated ROS formation in vHip in CUMS mice (Figure 2b). To determine the main cell types that undergo ferroptosis, we first counted neurons, microglia, and astrocytes in the vHip using immunofluorescence staining. Chronic stress induced a significant decrease in the number of astrocytes but not neurons or microglia (Figure 2c; Figure S2a, Supporting Information). Furthermore, chronic stress increased the number of TUNEL positive cells in the vHip of CUMS mice, predominantly in astrocytes (Figure 2d), rather than neurons or microglia (Figure S2b,c, Supporting Information). These results indicated that chronic stress promoted cell death and loss of astrocytes in vHip. Subsequently, the GPX4 expression in vHip was assessed. CUMS significantly reduced the number of GPX4‐positive cells, particularly GPX4‐positive astrocytes (GPX4^+^‐GFAP^+^/GFAP^+^) in vHip (Figure 2e), with consistent reductions observed across the dentate gyrus (DG), CA1 and CA3 subregions (Figure S3a–c, Supporting Information). In contrast, the GPX4 expression in neurons and microglia remained unchanged (Figure S3d,e, Supporting Information). The 2D Sholl analysis revealed that CUMS mice had markedly altered astrocyte morphology in vHip, characterized by shorter length, fewer branches, and reduced branch points and intersections (Figure 2f). TEM analysis further revealed that the mitochondria in the astrocytes of CUMS mice exhibited characteristic ferroptosis features, such as shrunken mitochondria, reduced mitochondrial cristae, and increased mitochondrial membrane density (Figure 2g). Since proteomics analysis revealed alterations in glutamatergic synapse in CUMS mice, we also examined synaptic changes in vHip neuron with TEM and Golgi‐cox staining. In contrast to the Ctrl mice, the CUMS mice exhibited notable decreases in synapse density and PSD thickness, although the synaptic cleft and PSD length were unaffected (Figure 2h). Golgi‐cox staining also demonstrated a decrease in dendritic spine count in the vHip of CUMS mice, mostly due to a lower proportion of branched spines, while other spine subtypes remained largely unchanged (Figure 2i).

Taken together, these results strongly suggest that chronic stress induced ferroptosis in astrocytes and impairments in neuronal synapses in vHip of mice.

### 2.3 Deferoxamine Ameliorates Depressive Behaviors via Inhibiting Astrocytic Ferroptosis

**Figure 3:**
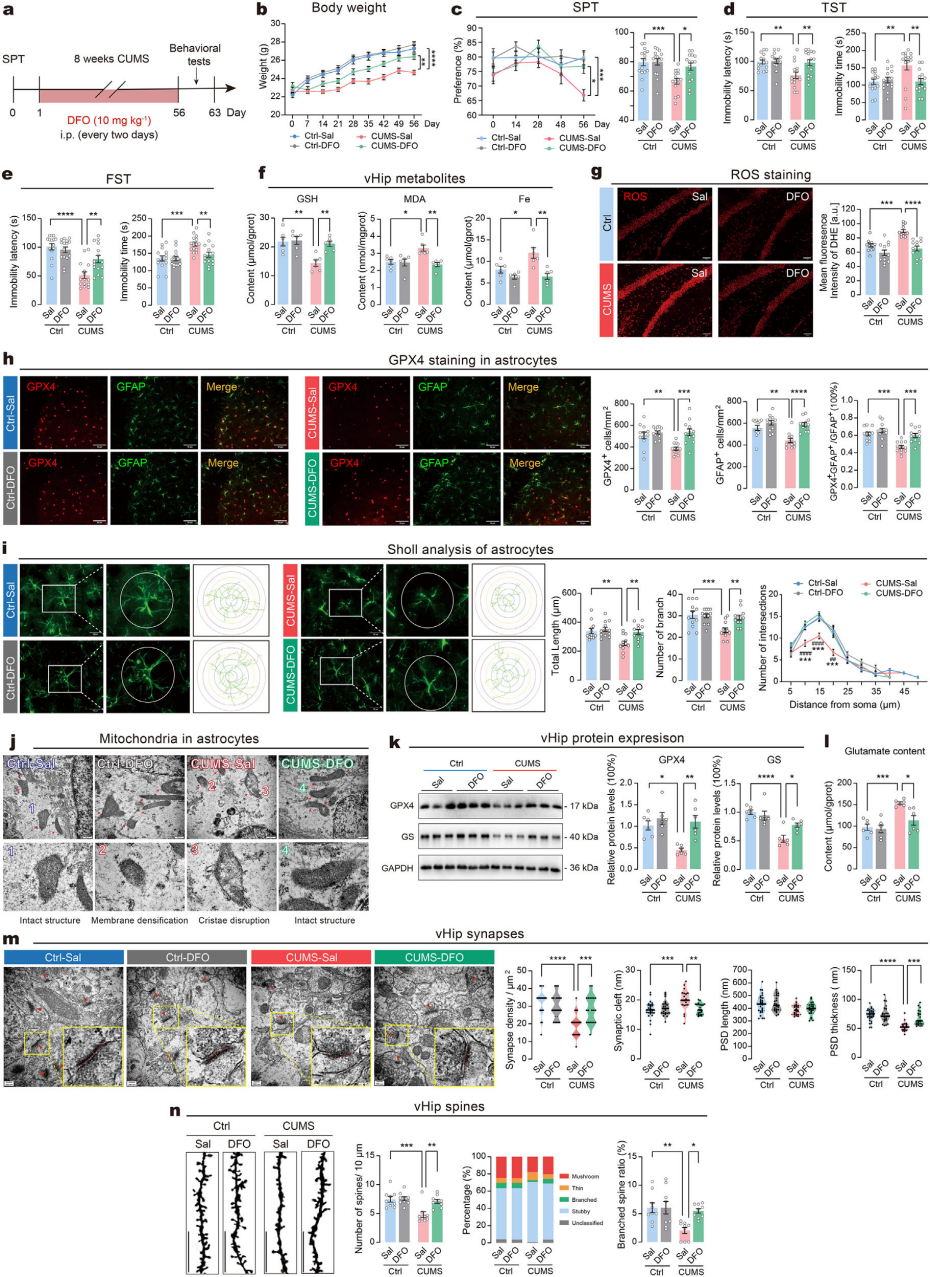
Deferoxamine ameliorates depressive behaviors via inhibiting astrocytic ferroptosis. a) Timeline of DFO intervention on CUMS‐induced depressive‐like behaviors in mice. *n* = 15 mice/group. b) Changes in body weight during CUMS. c) Sucrose preference in saline (Sal)‐ or DFO‐treated mice. d) Immobility latency and immobility time in TST. e) Immobility latency and immobility time in FST. f) Levels of GSH, MDA, and iron in vHip. *n* = 6 samples per group. g) Representative images (left) and quantification (right) of ROS immunoreactivity in vHip. Scale bar = 40 µm. *n* = 12 slices from 3 animals per group. h) Representative images (left) and quantification (right) of GPX4‐positive astrocytes in vHip. Scale bar = 50 µm. *n* = 12 slices from 3 animals per group. i) Analysis of astrocytic morphology in vHip. Left: Representative images of astrocytes. Right: Quantitative analysis of astrocytic morphology. Scale bar = 20 µm. *n* = 12 cells per group. j) Representative images of mitochondrial morphology in astrocytes captured by TEM. Mitochondria exhibited intact morphology and structure in Ctrl‐Sal group and CUMS‐DFO group (subpanel 1 and 4). The mitochondrial structure was unclear, with increased double membrane density and disrupted cristae in CUMS‐Sal group (subpanel 2 and 3). Scale bar = 500 nm. k) Left: Representative immunoblot images. Right: Expression levels of GPX4 and GS. *n* = 6 samples per group for analysis. l) The content of glutamate in vHip. *n* = 6 samples per group. m) Analysis of synaptic ultrastructure. Left: Representative TEM images. Right: Statistical analysis of synaptic ultrastructure. Scale bar = 200 nm. n) Morphological changes of dendritic spines in vHip using Golgi‐Cox staining. Representative images (left) and statistical analysis (right) of dendritic spines. Scale bars = 10 µm. Statistical analysis was performed using two‐way ANOVA followed by Tukey's post hoc test. Data are presented as means ± SEM. *P < 0.05, **P < 0.01, ***P < 0.001, ****P < 0.0001, ^##^ *P* < 0.01, ^####^ *P* < 0.0001.

Next, we tested whether ferroptosis in vHip astrocytes was responsible for depressive‐like behaviors induced by chronic stress. During the CUMS paradigm, the mice were administered with the ferroptosis inhibitor DFO (**Figure** **3**a). Treatment with DFO alone did not affect the body weight or depressive‐like behaviors in mice. However, in mice subjected to CUMS, DFO effectively countered weight loss (Figure 3b), restored sucrose preference (Figure 3c), shortened immobility time, and extended immobility latency in TST and FST (Figure 3d,e). No significant changes in locomotor activity were observed between groups (Figure S4a, Supporting Information). These results suggested that pharmacological inhibition of ferroptosis effectively ameliorated depressive‐like behaviors in CUMS mice. Next, we evaluated the ferroptosis‐related changes in vHip. Consistent with previous results, chronic stress increased vHip MDA content, Fe content and ROS production while decreasing GSH content, but all these changes were reversed by DFO treatment (Figure 3f,g). The results of immunostaining and TEM further revealed that DFO treatment alleviated ferroptosis in astrocytes, including restoring GPX4 expression (Figure 3h), recovering astrocyte morphology (Figure 3i), as well as preserving mitochondrial integrity (Figure 3j). Furthermore, the immunoblotting analysis verified that DFO treatment opposed the CUMS‐induced decreases in the expression of the ferroptosis marker GPX4 (Figure 3k). Interestingly, we also observed that DFO treatment reversed GS reduction in the CUMS group. Considering that GS is expressed primarily in astrocytes and is critical for extracellular glutamate clearance and neurotoxicity,^[^ ^8^, ^25^ ^]^ we assessed the glutamate content in vHip. We observed a significant increase in glutamate levels in the CUMS group, which were normalized by DFO treatment (Figure 3l). TEM analysis revealed that DFO treatment restored synaptic impairments in CUMS mice, including synaptic density, synaptic cleft, and PSD thickness, without affecting PSD length (Figure 3m). Furthermore, DFO prevented CUMS‐induced reduction in dendritic spine density and the proportion of branched spines (Figure 3n). These findings indicate that pharmacological inhibition of ferroptosis significantly alleviates stress‐induced depressive‐like behaviors, restores astrocyte function, and reverses glutamatergic neurotoxicity in vHip.

### 2.4 Ferroptosis in Astrocytic GL261 Cells Affects Glutamate Toxicity in Hippocampal HT22 Cells

**Figure 4:**
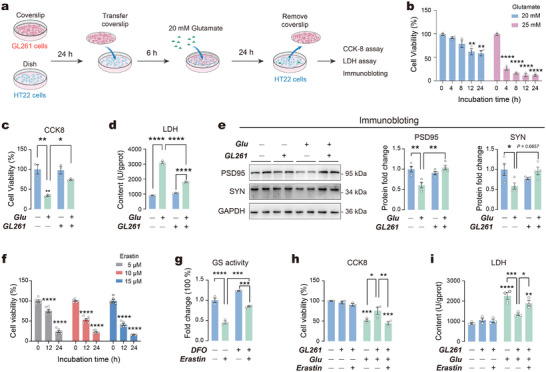
Ferroptosis in astrocytic GL261 cells affects glutamate toxicity in hippocampal HT22 cells. a) Schematic illustration of the glutamate treatment protocol in astrocyte‐neuron co‐cultures. b) Cell viability of HT22 cells following glutamate treatment (20–25 mm). sc) Cell viability with glutamate treatment in HT22 cells within co‐cultures. d) LDH levels with glutamate treatment in HT22 cells within co‐cultures. e) Protein expression levels in HT22 cells with glutamate treatment. Left: Representative immunoblot images. Right: Quantification of PSD95 and SYN expression levels in HT22 cells. f) Cell viability of GL261 cells following erastin (Era) treatment (5–15 µm). g) GS activity in GL261 cells after erastin treatment. h) Cell viability of HT22 cells under various experimental conditions. i) LDH levels in HT22 cells under various experimental conditions. *n* = 3–6 samples per group. Statistical analyses included one‐way ANOVA followed by Dunnett's post hoc test and two‐way ANOVA followed by Tukey's post hoc test. Data are presented as means ± SEM. *P < 0.05, **P < 0.01, ***P < 0.001, ****P < 0.0001.

The clearance of extracellular glutamate is mainly regulated by astrocytes.^[^ ^26^, ^27^ ^]^ Dysfunction in astrocytes may result in impaired glutamate clearance, which contributes to neuronal functional deficits and, when excessive, may lead to neuronal apoptosis.^[^ ^28^, ^29^ ^]^ Next, we established an in vitro cell co‐culture model (**Figure** **4**a) to explore how astrocyte ferroptosis impacts glutamate clearance and neurotoxicity in hippocampal cells. We performed the CCK‐8 assay to evaluate the cell toxicity at different glutamate concentrations and exposure durations on HT22 cells. In vitro, supraphysiological levels of glutamate are commonly used to mimic pathological accumulation in the absence of the brain's native microenvironment.^[^ ^30^, ^31^ ^]^ Our result revealed that glutamate exposure reduced viability in HT22 cells in a time‐dependent manner (Figure 4b). Specifically, after 24 h of exposure to 20 mm glutamate, the viability of HT22 cells dropped to 58.71%, which is consistent with previous studies,^[^ ^32^ ^]^ prompting us to select this concentration for subsequent experiments. Co‐culture with astrocytic GL261 cells significantly reduced glutamate toxicity in HT22 cells, as indicated by increased cell viability and decreased lactate dehydrogenase (LDH) levels (Figure 4c,d). Immunoblotting revealed that glutamate exposure significantly decreased the expression levels of the synaptic markers PSD95 and SYN. However, co‐culture with GL261 cells rescued the expression of PSD95 and SYN (*P* = 0.0657) in HT22 cells, suggesting a reduction in glutamate neurotoxicity (Figure 4e). Next, we applied erastin, a small molecule ferroptosis inducer, to GL261 cells to investigate the effects of ferroptosis on astrocytic functions. Erastin significantly decreased GL261 cell viability in a dose‐ and time‐dependent manner (Figure 4f). After a 12 h incubation with 10 µm erastin, the viability of GL261 cells decreased to 53.47%, leading to the selection of this concentration for later experiments. Glutamine synthetase (GS), a critical enzyme in the glutamate‐glutamine cycle, is essential for astrocyte‐mediated glutamate uptake and metabolism.^[^ ^33^ ^]^ We found that treatment with erastin significantly decreased GS activity in GL261 cells, which was further rescued by the ferroptosis inhibitor DFO (Figure 4g). Of particular significance, the protective capacity of GL261 cells against glutamate‐induced cytotoxicity in HT22 cells was effectively abrogated when GL261 cells were simultaneously exposed to erastin (Figure 4h,i). Collectively, these findings suggest that ferroptosis in astrocytic GL261 cells exacerbates glutamate toxicity in hippocampal HT22 cells.

### 2.5 PCBP1 is Responsible for Sensitivity of Astrocytic Ferroptosis and Susceptibility to Subthreshold Stress

**Figure 5:**
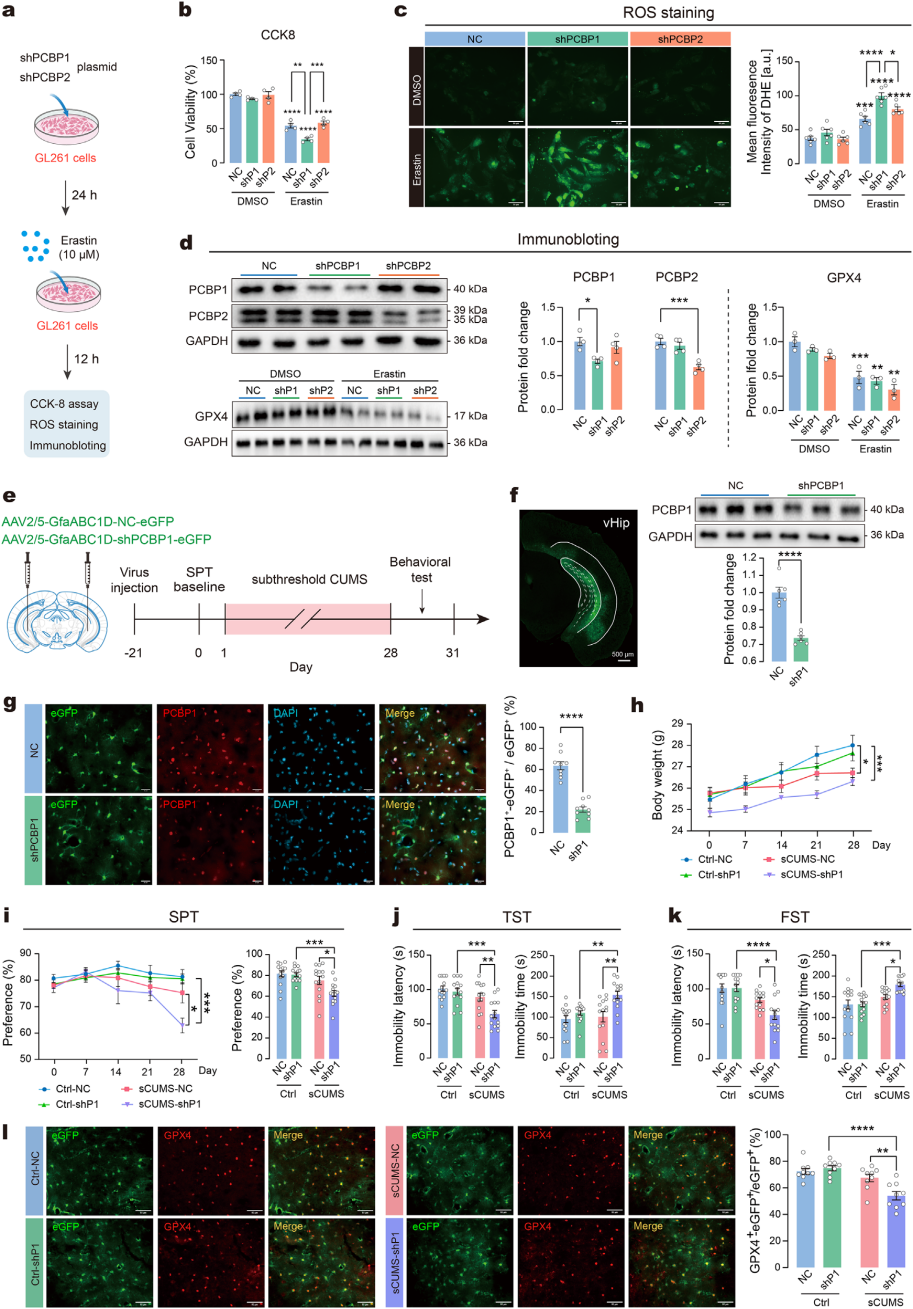
PCBP1 is responsible for sensitivity of astrocytic ferroptosis and susceptibility to subthreshold stress. a) Experimental workflow for erastin treatment in GL261 cells with shRNA‐expressing plasmids targeting PCBP1 (shP1) or PCBP2 (shP2). b) Cell viability following erastin treatment in GL261 cells. c) Representative images (left) and quantification (right) of DHE fluorescence indicating ROS levels in GL261 cells. Scale bars = 50 µm. d) Protein expression of PCBP1, PCBP2, and GPX4. Left: Representative immunoblot images. Right: Quantification of protein expression. *n* = 3–4 samples per group for cell experiments. e) Left: Schematic illustration of the viral injection site. Right: Timeline of shPCBP1 intervention in subthreshold CUMS (sCUMS)‐induced depressive‐like behaviors. f) Left: Representative coronal images of the injection site. Scale bars = 500 µm. Right: Representative images and quantification of PCBP1 expression in vHip. *n* = 6 samples per group. g) Representative images (left) and quantification (right) of PCBP1‐positive astrocytes in vHip. Scale bars = 50 µm. *n* = 9 slices from 3 animals per group. h) Body weight of mice during sCUMS. i) Sucrose preference of mice during sCUMS. j) Immobility latency and immobility time in TST. k) Immobility latency and immobility time in FST. *n* = 15 mice per group. l) Representative images (green, eGFP; red, GPX4) and quantification of the GPX4‐positive astrocytes in vHip. Scale bars = 50 µm. *n* = 9 slices from 3 animals per group. Statistical analyses included unpaired t‐test, one‐way ANOVA followed by Dunnett's post hoc test and two‐way ANOVA followed by Tukey's post hoc test. Data are presented as means ± SEM. *P < 0.05, **P < 0.01, ***P < 0.001, ****P < 0.0001.

Our next objective was to elucidate the cellular mechanisms that predispose vHip astrocytes to ferroptosis following chronic stress. In light of the phosphoproteomics results, which strongly suggested alterations in the expression of PCBP1 and PCBP2 within the ferroptosis pathway following chronic stress, we commenced an investigation to characterize the role of PCBP1 and PCBP2 in astrocyte ferroptosis in vitro. GL261 cells were transfected with plasmids expressing shRNA targeting either PCBP1 (shP1) or PCBP2 (shP2), followed by a 12 h exposure to 10 µm erastin, after which cell viability and ROS levels were measured (**Figure** **5**a). The results showed that knockdown of PCBP1 or PCBP2 alone had no effect on the cell viability and ROS levels in GL261 cells. However, to our surprise, knockdown of PCBP1, but not PCBP2, significantly exacerbated erastin‐induced cell death and increased ROS production compared to the negative control (NC) group (Figure 5b,c). As expected, after transfection with the shRNA plasmids, the protein expression of PCBP1 and PCBP2 showed specific reductions in the corresponding groups (Figure 5d). In addition, the expression level of GPX4 did not differ significantly among the groups following erastin exposure. Similar experiments in C8‐D1A astrocytes showed consistent results. PCBP1 knockdown significantly exacerbated erastin‐induced cell death and increased ROS levels, while GPX4 expression remained unchanged among the groups after erastin treatment (Figure S5a–c, Supporting Information). These results indicate that knockdown of PCBP1 may sensitize astrocytes to erastin‐induced ferroptosis in vitro and prompt us to investigate whether downregulation of PCBP1 could affect behavioral susceptibility to chronic stress in vivo.

Considering that susceptibility to chronic stress is a crucial factor for depression, we used a subthreshold CUMS (sCUMS) paradigm^[^ ^34^ ^]^ to explore how PCBP1 knockdown in vHip astrocytes affects depressive‐like behaviors in mice. Three weeks before the sCUMS protocol, AAVs expressing astrocyte‐specific PCBP1 shRNA were bilaterally injected into the vHip of mice (Figure 5e). The downregulation of PCBP1 protein in vHip was confirmed by immunoblotting (Figure 5f). To verify the knockdown efficiency and cellular specificity, triple immunofluorescence staining was performed (Figure S6a, Supporting Information). Quantitative analysis revealed that 87.02 ± 1.10% and 87.49 ± 1.07% of GFAP⁺ astrocytes expressed eGFP in NC and shP1 groups, respectively (Figure S6b,c, Supporting Information), confirming high and consistent transduction efficiency across groups. Further co‐localization analysis of PCBP1 and eGFP confirmed efficient PCBP1 knockdown in eGFP⁺ astrocytes within vHip (Figure 5g). Depressive‐like behaviors in mice were evaluated after the last sCUMS procedure. Compared to the Ctrl group, PCBP1 knockdown in vHip astrocytes alone did not cause alterations in body weight, while subthreshold stress caused a notable reduction in body weight of mice in the sCUMS groups (Figure 5h). In SPT, subthreshold stress or PCBP1 knockdown in vHip astrocytes alone did not affect sucrose preference. However, PCBP1 knockdown notably lowered the sucrose preference in mice exposed to subthreshold stress (Figure 5i). Moreover, the shPCBP1 mice subjected to subthreshold stress exhibited reduced immobility latency and prolonged immobility time in both TST and FST, in contrast to the absence of such effects in the NC group (Figure 5j,k). No significant differences in locomotor activity were found between the groups (Figure S4b, Supporting Information). To further validate the role of PCBP1 in regulating astrocytic ferroptosis in vivo, we measured ferroptosis markers in vHip following astrocytic PCBP1 knockdown. sCUMS or PCBP1 knockdown alone did not significantly alter the proportion of GPX4^+^ astrocytes (GPX4^+^‐eGFP^+^/eGFP^+^ cells) or GSH and MDA levels. However, PCBP1 knockdown combined with sCUMS significantly reduced astrocytic GPX4 expression (Figure 5l), decreased GSH levels, and increased MDA levels (Figure S6d,e, Supporting Information). Collectively, these findings indicate that PCBP1 influences the sensitivity to ferroptosis in astrocytes, as well as the susceptibility to subthreshold stress in mice.

### 2.6 Astrocytic PCBP1 Improves Chronic Stress‐Induced Depressive‐Like Behaviors

**Figure 6:**
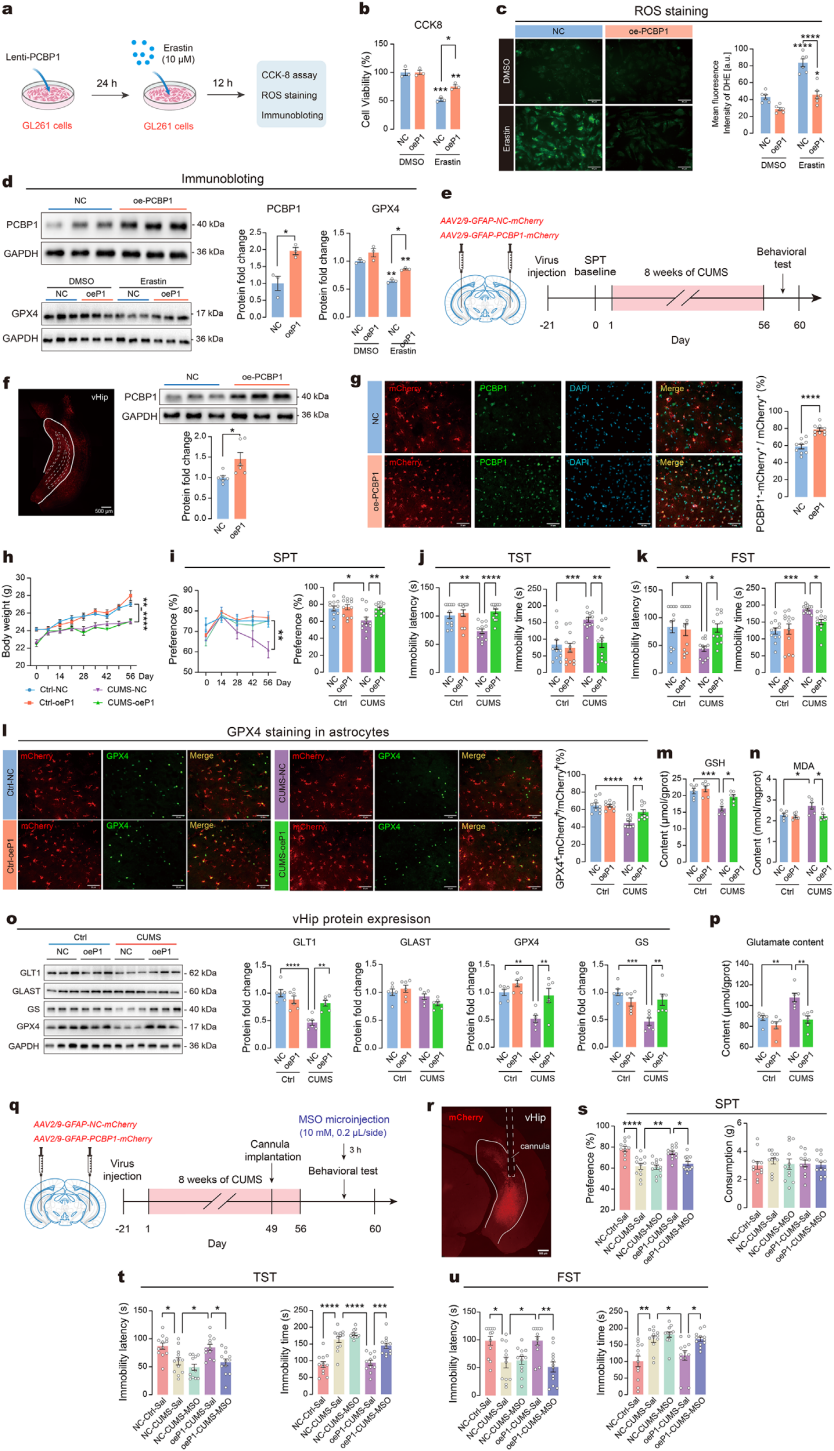
Astrocytic PCBP1 protects astrocyte from ferroptosis and improves chronic stress‐induced depressive‐like behaviors. a) Experimental workflow for erastin treatment in GL261 cells with PCBP1 overexpression (oeP1). b) Cell viability following erastin treatment in GL261 cells. c) ROS fluorescence quantified by H2DCFDA. Representative DHE staining images (left) and quantification (right) in GL261 cells. Scale bar = 50 µm. d) Left: Representative immunoblot images. Right: Quantification of protein expression of PCBP1 and GPX4 in GL261 cells. *n* = 3–4 samples per group for cell experiments. e) Experimental procedure of CUMS regime with astrocytic PCBP1 overexpression in vHip. Left: Schematic illustration of the viral injection site. Right: Experimental timeline. f) Left: Representative images showing virus expression (mCherry) in vHip. Scale bar = 500 µm. Right: Representative images and quantification of PCBP1 expression in vHip. *n* = 6 samples per group. g) Representative images (left) and quantification (right) of PCBP1‐positive astrocytes in vHip. Scale bars = 50 µm. *n* = 9 slices from 3 animals per group. h) Changes in body weight of mice. i) Sucrose preference of mice during CUMS. j) Immobility latency and immobility time in TST. k) Immobility latency and immobility time in FST. *n* = 12 mice/group. l) Representative images (red, mCherry; green, GPX4) and quantification of the GPX4‐positive astrocytes in vHip. Scale bars = 50 µm. *n* = 9 slices from 3 animals per group. m,n) Levels of GSH (m) and MDA (n) in vHip. *n* = 6 samples per group. o) Left: Representative immunoblot images. Right: Expression levels of GLT1, GLAST, GPX4 and GS. *n* = 6 samples/group. p) Glutamate content in vHip. *n* = 6 samples per group. q) Experimental procedure of CUMS regime with MSO treatment in vHip. Left: Schematic illustration of the viral injection site and cannula implantation. Right: Experimental timeline. r) Representative images showing cannula implantation. Scale bar = 500 µm. s) Sucrose preference of mice. t) Immobility latency and immobility time in TST. u) Immobility latency and immobility time in FST. *n* = 12 mice per group. Statistical analyses included unpaired t‐test, one‐way/two‐way ANOVA followed by Tukey's post hoc test. Data are presented as means ± SEM. *P < 0.05, **P < 0.01, ***P < 0.001, ****P < 0.0001.

We next aimed to investigate whether upregulation of PCBP1 is sufficient to counteract erastin‐induced ferroptosis in astrocytes in vitro. Astrocytic GL261 cells were infected with lentivirus overexpressing PCBP1 and incubated with the ferroptosis inducer erastin for 12 h, after which cell viability and ROS levels were measured (**Figure** **6**a). The results showed that overexpression of PCBP1 (oeP1) alone had no effect on cell viability or ROS levels in GL261 cells. Nonetheless, when PCBP1 was overexpressed, it protected GL261 cells from erastin‐induced cell death and decreased ROS levels in comparison to the negative control (NC) group (Figure 6b,c). As expected, the protein level of PCBP1 showed a significant elevation in the oeP1 group (Figure 6d). Furthermore, while the overexpression of PCBP1 alone did not alter GPX4 expression levels, it led to an increase in GPX4 expression in the group treated with erastin. These protective effects were confirmed in C8‐D1A astrocytes, where PCBP1 overexpression similarly enhanced ferroptosis resistance, with improved cell viability, decreased ROS levels, and restoration of GPX4 expression (Figure S5a–c, Supporting Information). These results suggest that upregulation of PCBP1 could protect astrocytes from erastin‐induced ferroptosis in vitro.

Given that downregulation of PCBP1 increases the susceptibility to subthreshold stress in mice, we next asked whether upregulation of PCBP1 in vHip astrocytes could prevent chronic stress‐induced depressive‐like behaviors. Three weeks before starting the CUMS protocol, AAVs designed to overexpress PCBP1 specifically in astrocytes were bilaterally injected into the vHip of mice (Figure 6e). The upregulation of PCBP1 protein in vHip was confirmed with immunoblotting (Figure 6f). Triple immunofluorescent staining was performed to verify the efficiency and cellular specificity of viral transduction (Figure S7a, Supporting Information). Quantitative analysis showed that 89.64 ± 1.33% (NC group) and 87.90 ± 1.33% (oeP1 group) of GFAP⁺ astrocytes expressed mCherry (Figure S7b, Supporting Information), indicating high and consistent transduction efficiency across groups. Further co‐localization analysis of PCBP1 and mCherry confirmed efficient PCBP1 overexpression in mCherry⁺ astrocytes within vHip (Figure 6g). After the final CUMS procedure, depressive‐like behaviors in mice were assessed. Overexpression of PCBP1 in vHip astrocytes did not lead to any changes in the body weight of mice in either the Ctrl or CUMS group (Figure 6h). In SPT, while overexpression of PCBP1 in vHip astrocytes alone did not affect sucrose preference, it notably increased the sucrose preference in mice exposed to CUMS (Figure 6i). Furthermore, overexpression of PCBP1 significantly increased the immobility latency and shortened the immobility time in mice from the CUMS group during both TST and FST, while no such effects were observed in the NC group (Figure 6j,k). No significant differences in locomotor activity were found between the groups (Figure S4c, Supporting Information). To further verify the in vivo effects of overexpressing PCBP1 in astrocytes on ferroptosis and glutamate homeostasis, we next examined ferroptosis markers and key proteins involved in glutamate metabolism. PCBP1 overexpression significantly reversed the CUMS‐induced reduction in the proportion of GPX4^+^ astrocytes (GPX4^+^‐mCherry^+^/mCherry^+^), restored GSH levels, and attenuated MDA accumulation in vHip (Figure 6l–n). Western blot analysis showed that PCBP1 overexpression reversed CUMS‐induced reduction in GPX4, GLT1, and GS, while GLAST levels remained unchanged (Figure 6o). Moreover, PCBP1 overexpression markedly decreased glutamate content (Figure 6p).

To clarify whether PCBP1 exerts antidepressant effects through regulating glutamate homeostasis, we administered the GS inhibitor MSO into the vHip 3 h prior to behavioral testing. MSO treatment completely reversed the behavioral improvements conferred by PCBP1 overexpression in CUMS mice, as evidenced by significantly reduced sucrose preference (Figure 6s) and increased immobility time in both TST (Figure 6t) and FST (Figure 6u), reaching levels comparable to those of CUMS control groups. Notably, MSO did not exacerbate depressive‐like behaviors in CUMS mice without PCBP1 overexpression. No significant differences in locomotor activity were found between the groups (Figure S4d, Supporting Information). These findings provide causal evidence that PCBP1's antidepressant effects are mediated through glutamate homeostasis in vHip astrocytes.

Collectively, these results demonstrate that astrocytic PCBP1 upregulation effectively inhibits ferroptosis, restores glutamate homeostasis, and ameliorates CUMS‐induced depressive‐like behaviors.

### 2.7 Astrocytic PCBP1 Enhances Glutamatergic Neuronal Activity and Neurophysiology

**Figure 7:**
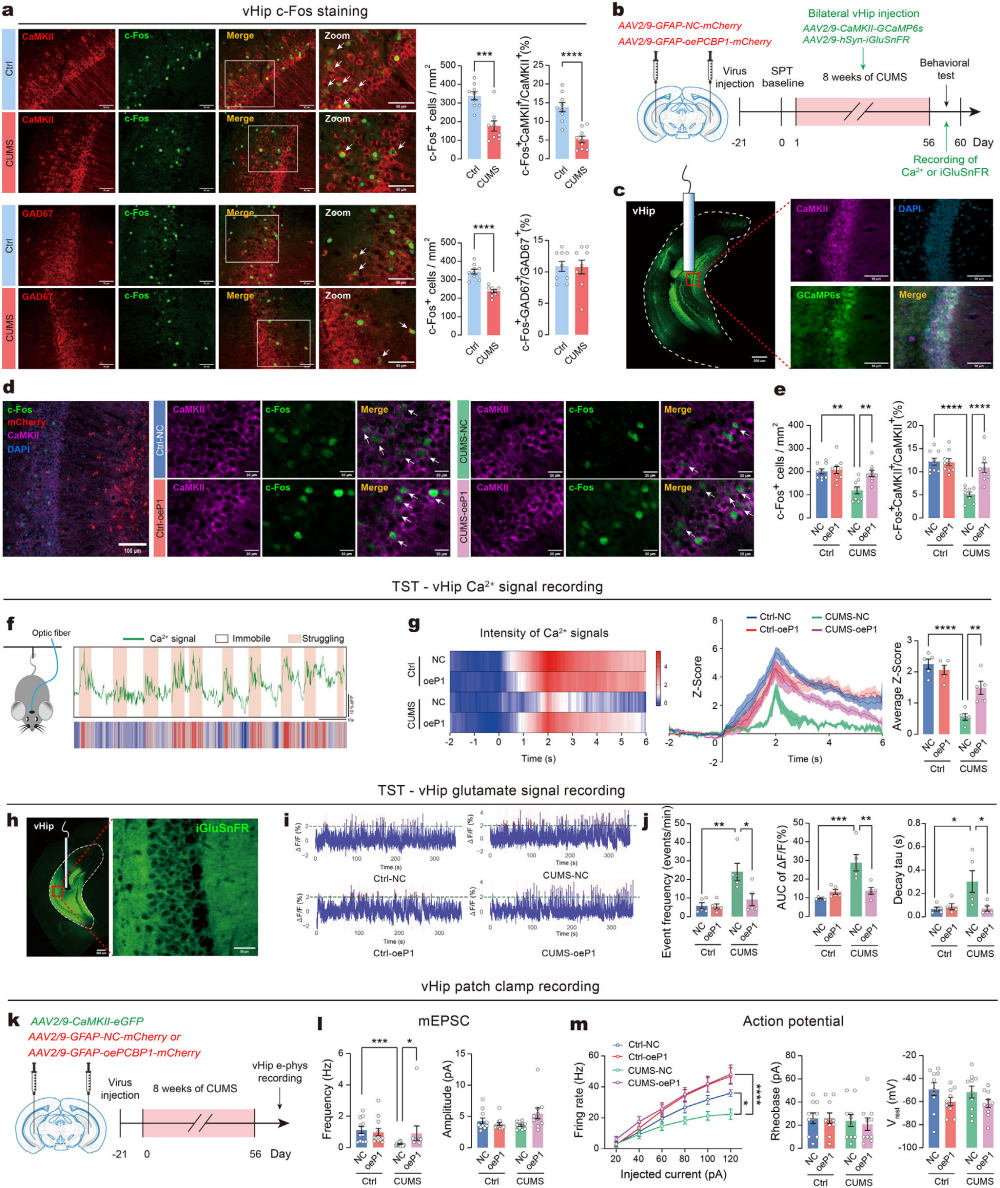
Astrocytic PCBP1 enhances glutamatergic neuronal activity and neurophysiology. a) c‐Fos expression in vHip of mice following CUMS. Above: Representative images and quantification of c‐Fos‐positive glutamatergic neurons. Below: Representative images and quantification of c‐Fos‐positive GABAergic neurons. Scale bars = 50 µm. *n* = 9 slices from 3 animals per group. b) Timeline of fiber photometry recording Ca^2+^ and glutamate signals from vHip c) Left: Representative images validate GCaMP6s expression in vHip. Scale bar = 500 µm. Right: Representative images showing the overlap between GCaMP6s‐expressing cells (green) and glutamatergic neurons (violet). Scale bar = 50 µm. d) Representative images of c‐Fos‐positive glutamatergic neurons. Scale bars = 100 or 20 µm. e) Statistical analysis of c‐Fos co‐labeled glutamatergic neuron in vHip of mice. f) Schematic of fiber photometry recording in TST and representative Ca^2+^ signals photometric traces in response to the TST during struggling and immobility phases. Scale bars = 10 s. g) Ca^2^⁺ signal recording in vHip during TST. Left: Representative heatmaps showing Ca^2^⁺ signal in response to TST during struggling. Right: Z‐score traces and quantification of average Z‐scores during TST. *n* = 5 mice per group. h) Representative images of the AAV vectors engineered to express the glutamate sensor iGluSnFR under the hSyn promoter in the vHip. Scale bars = 500 µm / 50 µm. i) Representative trace of glutamate signal event detection during TST. Red dots indicate detected events. j) Event‐based quantification. Event frequency (left), AUC (middle) and decay tau (right). *n* = 5 mice per group. k) Timeline of electrophysiological recordings from vHip glutamatergic neurons. l) Quantification of mEPSC frequency (left) and amplitude (right) recorded from vHip glutamatergic neurons. *n* = 10–12 cells from 3 mice per group. m) Left: Evoked firing rates of action potentials recorded from eGFP⁺ neurons in the vHip. Right: Quantifications of rheobase and resting membrane potential recorded from eGFP^+^ neurons in vHip. *n* = 10 cells from 3 mice per group. Statistical analyses included unpaired t‐test, one‐way/two‐way ANOVA followed by Tukey's post hoc test and Kruskal‐Wallis test. Data are presented as means ± SEM. *P < 0.05, **P < 0.01, ***P < 0.001, ****P < 0.0001.

Based on our previous findings that ferroptosis in astrocytic GL261 cells exacerbated glutamate toxicity in hippocampal HT22 cells, we next investigated whether PCBP1‐mediated suppression of ferroptosis in astrocytes could mitigate chronic stress‐induced functional impairments in vHip neurons. To validate the responsiveness to stress in vHip neurons, immunostaining of c‐Fos, an immediate‐early gene marker indicative of neuronal activity, was performed in mice from both the Ctrl and CUMS groups immediately after the final FST. Forced swimming induced robust c‐Fos expression in the vHip of Ctrl mice, but CUMS mice showed reduced c‐Fos expression, indicating decreased neuronal responsiveness to stress in these mice. Further analysis revealed a significant decrease in the co‐localization ratio of c‐Fos with CaMKII‐positive cells, but not with GAD67‐positive cells (**Figure** **7**a), suggesting that the hippocampal cells primarily responsible for differential stress responses are glutamatergic excitatory neurons, rather than GABAergic inhibitory neurons. We then investigated whether astrocytic PCBP1 could influence glutamatergic neuron activity in the vHip after chronic stress. Using AAV microinjection, we overexpressed PCBP1 in vHip astrocytes and subsequently employed fiber photometry to record glutamatergic neuronal activity in the vHip of CUMS mice with GCaMP6s expression during TST and FST (Figure 7b). Confocal images confirmed that GCaMP6s was specifically expressed in CaMKII‐positive cells in vHip (Figure 7c). By immunostaining, we found that astrocytic PCBP1 overexpression increased both the total number of c‐Fos‐positive cells and their proportion among glutamatergic neurons in vHip of CUMS mice (Figure 7d,e). We then used fiber photometry recording of GCaMP6s‐expressing vHip glutamatergic neuronal activity in TST. GCaMP6s‐expressing mice exhibited robust calcium signal fluctuations (Figure S8a,b, Supporting Information), which were absent in eGFP‐expressing controls (Figure S8c,d, Supporting Information), confirming that these signals were calcium‐dependent rather than motion artifacts. The recordings showed a pattern in which mice exhibiting motivated escape behavior alternated between passive periods of immobility (associated with low calcium signals) and active periods during struggling (associated with high calcium signals in neurons) (Figure 7f). We evaluated the intensity of calcium signals within six seconds of a randomly initiated struggle event in mice. CUMS mice exhibited a noticeably reduced calcium signal intensity compared to Ctrl mice. Nonetheless, in mice with astrocytic PCBP1 overexpression, the pattern of calcium signal intensity resembled that of the Ctrl group (Figure 7g). In FST, the calcium signal peaks induced by struggle mirrored those observed in TST (Figure S8f, Supporting Information). After CUMS, mice with astrocytic PCBP1 overexpression displayed significantly higher calcium signal intensity during struggling behavior compared to those in the NC group (Figure S8g–i, Supporting Information). These results suggest that astrocytic overexpression of PCBP1 rescues the CUMS‐induced reduction in calcium activity of vHip glutamatergic neurons.

To determine whether the astrocytic PCBP1 modulates dynamic changes in glutamate levels in the vHip, we employed the glutamate sensor iGluSnFR driven by the hSyn promoter (Figure 7b,h). iGluSnFR‐expressing mice showed robust glutamate signal fluctuations during TST (Figure S8j,k, Supporting Information), which were absent in eGFP‐expressing controls (Figure S8l,m, Supporting Information), confirming the signal specificity. Event‐based analysis of glutamate transients revealed that CUMS mice exhibited significantly increased event frequency, larger area under the curve (AUC), and a prolonged decay time constant compared to Ctrl mice in TST (Figure 7i,j) and FST (Figure S8n,o, Supporting Information). Importantly, astrocytic PCBP1 overexpression normalized these CUMS‐induced alterations in glutamate dynamics to Ctrl levels in the vHip. Next, we conducted the whole‐cell patch‐clamp recordings in vHip slices to evaluate the activity of glutamatergic neurons (Figure 7k). CUMS significantly decreased the frequency of mEPSC (Figure 7l; Figure S8p,q, Supporting Information) and the firing rate of evoked action potentials (Figure 7m; Figure S8r, Supporting Information) in glutamatergic neurons. However, these neuronal impairments were reversed by astrocytic PCBP1 overexpression.

Together, we demonstrate that the hypoactivity of glutamatergic neurons and glutamate dysregulation induced by chronic stress are effectively reversed by PCBP1 overexpression in astrocytes of the vHip.

### 2.8 Potential Mechanisms for PCBP1‐Regulated Astrocytic Ferroptosis Sensitivity

**Figure 8:**
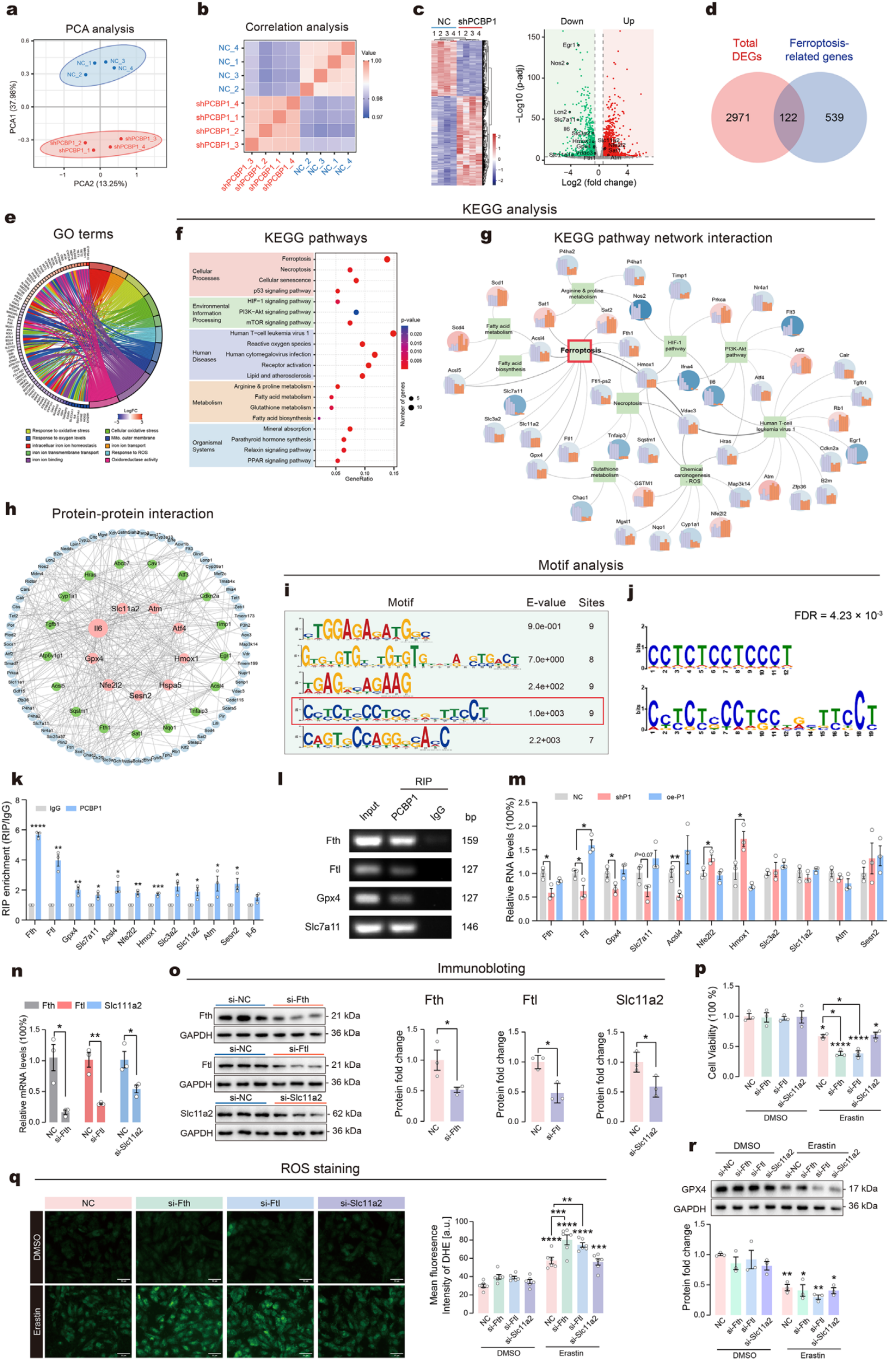
Potential mechanisms for PCBP1‐regulated astrocytic ferroptosis sensitivity. a) PCA of transcriptomic profiles in GL261 cells. b) Correlation heatmap showing relationships between the NC and shPCBP1 groups. c) Heatmap (left) and volcano map (right) showing DEGs between shPCBP1 and NC groups. d) Venn diagram between total DEGs and ferroptosis‐related genes extracted from databases. e) GO enrichment analysis highlighting terms related to cellular iron ion balance and oxidative stress. f) Dot plot showing the enriched KEGG pathways. g) C‐net plot illustrating the genes involved in enriched KEGG pathways and their interactions. h) The protein–protein interactions (PPI) network among 102 ferroptosis‐related DEGs. i) Top five enriched sequences identified via motif analysis of the nine key hub genes. j) The cytosine‐rich motif significantly matches the known PCBP1 binding motif. k) Relative enrichment of PCBP1‐bound mRNAs in GL261 cells as assessed by RIP‐qPCR. l) PCR gel showing enrichment of Fth, Ftl, Gpx4, and Slc7a11 mRNAs in PCBP1 immunoprecipitates. IgG served as a negative control. m) Quantitative RT‐PCR analysis of mRNA levels in GL261 cells with knockdown or overexpression of endogenous PCBP1. n) Quantitative RT‐PCR analysis of mRNA levels in GL261 cells with knockdown of Fth, Ftl, and Slc11a2. o) Representative images and quantification of Fth, Ftl, and Slc11a2 expression in GL261 cells after siRNA interference. *n* = 3 samples per group. p) Cell viability following erastin treatment in GL261 cells. q) Representative images (left) and quantification (right) of DHE fluorescence indicating ROS levels in GL261 cells. Scale bars = 50 µm. r) Representative images (above) and quantification (below) of GPX4 expression. *n* = 3 samples per group for cell experiments. Statistical analyses included unpaired t‐test, Mann‐Whitney test and two‐way ANOVA followed by Tukey's post hoc test. Data are presented as means ± SEM. *P < 0.05, **P < 0.01, ***P < 0.001, ****P < 0.0001.

To further investigate the mechanisms by which PCBP1 regulates ferroptosis in astrocytes, we transfected GL261 cells with shPCBP1 plasmids to silence PCBP1 expression, followed by transcriptomic analysis. PCA revealed that the first principal component (PC1, 37.98%) distinctly separated the NC and shPCBP1 groups, indicating marked transcriptional divergence (**Figure** **8**a). High accuracy and reproducibility were confirmed by Pearson correlation coefficients exceeding 0.97 among replicates (Figure 8b). A total of 3093 differentially expressed genes (DEGs) were identified, including 1786 upregulated and 1307 downregulated genes (Figure 8c; also see Table S3A, Supporting Information). KEGG enrichment analysis of all DEGs revealed significant involvement in apoptosis, ferroptosis, and oxidative stress‐related pathways (Table S4A, Supporting Information), suggesting that PCBP1 knockdown may affect multiple cellular pathways. We next focused on ferroptosis‐related DEGs by cross‐referencing the 3093 DEGs with 661 ferroptosis‐associated genes sourced from the FerrDB, KEGG, and AmiGO2 databases, identifying 122 overlapping DEGs (Figure 8d; also see Table S3B, Supporting Information). To further investigate the functional implications of the ferroptosis‐related DEGs, GO and KEGG enrichment analyses were performed on the 122 DEGs. GO analysis revealed significant enrichment in terms related to oxidative stress, responses to oxygen levels, mitochondrial outer membrane, and intracellular iron ion homeostasis, indicating that PCBP1 downregulation significantly disrupts cellular iron balance and oxidative stress in astrocytes (Figure 8e; also see Table S4B, Supporting Information). KEGG analysis showed that these DEGs were primarily enriched in pathways associated with ferroptosis, necroptosis, HIF‐1 signaling, reactive oxygen species, fatty acid metabolism, glutathione metabolism, and mineral absorption (Figure 8f; also see Table S4C, Supporting Information). An interaction network among key enriched KEGG pathways was depicted in Figure 8g, highlighting DEGs closely linked to ferroptosis signaling, including Sat1, Sat2, Acsl4, Acsl5, Fth1, Slc7a11, Slc3a2, Slc11a2, Gpx4, Ftl1, Ftl‐ps2, Hmox1, and Vdac3. We next performed the protein‐protein interaction (PPI) analysis to explore the relationships among the 122 ferroptosis‐related DEGs, where nodes were color‐coded as red for central hub genes, green for significant interactors, and blue for peripheral genes (Figure 8h). Nine key hub genes, including Il6, Slc11a2, Atm, Atf4, Hmox1, Hspa5, Sesn2, Nfe2l2, and Gpx4, demonstrated significant connectivity, indicating their pivotal roles within the network.

Numerous studies have shown that PCBP1 binds to cytosine‐rich motifs in gene promoters to regulate transcription.^[^ ^35^, ^36^ ^]^ To investigate whether PCBP1 influences the mRNA expression of identified hub genes, we searched for PCBP1‐binding motifs within 1‐kb upstream using the MEME suite and identified the five most significantly enriched motifs (Figure 8i). Using the TomTom tool, we found that the cytosine‐rich motif identified from hub gene upstream regions significantly matched the known PCBP1 binding motif (FDR = 4.23 × 10^−3^), with a 12‐bp overlap and highly conserved sequence (Figure 8j). These results suggest that PCBP1 binding regulates the mRNA abundance of these hub genes. In summary, our transcriptomic analysis reveals that PCBP1 regulates ferroptosis sensitivity through modulation of genes involved in oxidative stress and iron homeostasis, highlighting the critical role of PCBP1 in the ferroptosis pathway.

To validate whether PCBP1 directly regulates ferroptosis‐related targets identified by transcriptomic analysis, we performed RIP‐qPCR in GL261 cells. Among the 12 selected genes (Fth, Ftl, Gpx4, Slc7a11, Acsl4, Nfe2l2, Hmox1, Slc3a2, Slc11a2, Atm, Sesn2, and Il6), 11 showed significant enrichment in PCBP1 immunoprecipitates, suggesting potential protein‐mRNA binding (Figure 8k). Representative PCR products amplified from RIP‐derived cDNA were confirmed by agarose gel electrophoresis (Figure 8l). Further expression analysis following PCBP1 knockdown or overexpression revealed that only Ftl exhibited consistent bidirectional regulation, being downregulated upon PCBP1 silencing and upregulated upon PCBP1 overexpression (Figure 8m). To functionally validate PCBP1‐bound targets, we performed siRNA‐mediated knockdown of three iron homeostasis‐related genes, Ftl, Fth, and Slc11a2. Efficient knockdown at both mRNA and protein levels was confirmed (Figure 8n,o). Upon erastin treatment, Ftl or Fth silencing significantly enhanced susceptibility to ferroptosis, as evidenced by increased cell death and elevated intracellular ROS levels, whereas Slc11a2 knockdown had no effect (Figure 8p,q). Additionally, erastin treatment substantially reduced GPX4 expression across all knockdown groups (Figure 8r). Collectively, these findings indicate that Ftl serves as the functionally dominant downstream target by which PCBP1 modulates astrocytic ferroptosis sensitivity.

## 3 Discussion

In this study, we used a mouse model to explore the pathophysiological mechanisms of depression. Combined proteomic and phosphoproteomic analyses of the vHip identified stress‐induced molecular changes, with enrichment analyses suggesting a potential involvement of the ferroptosis pathway. Subsequent validation experiments confirmed that CUMS‐induced depressive‐like behaviors were indeed associated with astrocytic ferroptosis in vHip. Further experiments showed that downregulation of astrocytic PCBP1 contributed to ferroptosis and depressive phenotype by disrupting glutamate homeostasis, leading to glutamate accumulation and glutamatergic neuronal toxicity. In contrast, PCBP1 overexpression in astrocytes prevented ferroptosis and CUMS‐induced damage in vHip glutamatergic neurons, and alleviated depressive‐like behaviors through a mechanism mediating glutamate clearance, as demonstrated by MSO rescue experiments. These findings implicate astrocyte‐specific ferroptosis as a novel contributor to depression pathogenesis. By delineating its cell‐type specificity, molecular regulation, and regional selectivity, our study refines the understanding of stress‐induced depression. Notably, PCBP1‐regulated ferroptosis in vHip astrocytes may serve as a promising therapeutic target for chronic stress‐related depression.

Astrocytes, characterized by their radial morphology and long fiber‐like projections that form gap junctions with surrounding cells, are essential for regulating blood flow, recycling glutamate, promoting synaptogenesis, and supporting various other brain functions.^[^ ^37^, ^38^ ^]^ Consistent with previous studies,^[^ ^39^ ^]^ our results suggested that stress significantly reduced the number of astrocytes and induced morphological atrophy in vHip. However, the exact mechanism underlying these stress‐induced changes remains unclear. As a key regulator of ferroptosis, GPX4 protects cell membranes from oxidative damage by reducing lipid peroxidation and lowering ROS levels.^[^ ^40^ ^]^ In particular, we observed ferroptosis‐related pathological changes in the vHip of CUMS mice. Furthermore, chronic stress induced decreases in GPX4 expression and mitochondrial damage in astrocytes, which were reversed by the ferroptosis inhibitor DFO, confirming that chronic stress induced astrocytic ferroptosis in vHip. Importantly, DFO treatment not only rescued astrocytic structural and molecular abnormalities but also significantly improved depressive‐like behaviors, suggesting that inhibition of astrocytic ferroptosis is involved in the antidepressant effects of DFO.

Phosphoproteomic analysis identified markedly decreased levels of phosphorylated PCBP1 and PCBP2, which are strongly associated with the ferroptosis signaling pathway. Based on these findings, we hypothesize that PCBP1 and PCBP2 play crucial roles in stress‐induced astrocytic ferroptosis in vHip. The PCBP family comprises hnRNP K and PCBP 1–4, with PCBP1 and PCBP2 sharing 89% amino acid sequence similarity and being abundantly expressed in the brain. Studies have indicated that PCBP1 and PCBP2 are involved in mRNA stabilization, translational regulation, and protection against ferroptosis through iron chaperone activity.^[^ ^16^ ^]^ Ferroptosis, a newly recognized type of cell death, is triggered by iron‐dependent lipid peroxidation.^[^ ^41^ ^]^ PCBP1 and PCBP2 protect against ferroptosis by chelating ferrous iron and reducing its oxidative activity in the cytoplasm, and their phosphorylation modulates subcellular localization and functional activities.^[^ ^36^, ^42^ ^]^ Due to the lack of highly specific commercial antibodies against phosphorylated PCBP1 and PCBP2, we employed the Phos‐tag SDS‐PAG to examine the phosphorylation change in these proteins. The results verified the decreased phosphorylation levels of PCBP1 and PCBP2 in vHip of CUMS mice, highlighting their pivotal role in stress‐induced ferroptosis. However, research on the upstream kinases regulating PCBP1 and PCBP2 phosphorylation remains limited and inconclusive. While PAK1 and AKT2 have been identified as PCBP1 kinases,^[^ ^36^, ^43^ ^]^ the specific kinases mediating stress‐induced phosphorylation changes in the brain remain unidentified, and no specific kinases for PCBP2 have been reported. This knowledge gap has led to a lack of effective genetic and pharmacological tools for phosphorylation‐specific modulation of these proteins in vivo. Therefore, we shifted our focus toward investigating the effects of modulating PCBP1 and PCBP2 expression levels on stress‐induced astrocytic ferroptosis and its implications for the depressive phenotype. Whether PCBP1 or PCBP2 regulates cellular sensitivity to ferroptosis remains controversial.^[^ ^17^, ^44^ ^]^ It is important to note that in our study there were no differences in the expression levels of PCBP1 and PCBP2 between the CUMS and control groups. The proteomic analysis encompasses the entire vHip, including various cell types such as microglia, neurons, astrocytes, oligodendrocytes, and others. Consequently, the sequencing data reflect the overall protein expression across all tissues and cell types. Thus, an inherent limitation that we cannot distinguish protein level changes at the cellular level by proteomics sequencing results, such as PCBP1 and PCBP2 alterations in astrocytes. Therefore, we utilized knockdown and overexpression techniques on astrocytes in vitro and highlighted the critical role of PCBP1 in erastin‐induced ferroptosis within GL261 astrocytes and C8‐D1A astrocytes. Knockdown of PCBP1, but not PCBP2, sensitized astrocytes to erastin‐induced ferroptosis, whereas PCBP1 overexpression effectively suppressed it. Although PCBP1 and PCBP2 share high homology, their divergent roles in ferroptosis regulation may be attributed to distinct subcellular localization and downstream targets in iron metabolism. PCBP1 localizes to mitochondria and iron‐rich compartments,^[^ ^45^ ^]^ while PCBP2 is recruited to P‐bodies and stress granules.^[^ ^46^ ^]^ Functionally, PCBP1 maintains labile iron pool (LIP) homeostasis via ferritin binding and ferritinophagy suppression, which is a key determinant of ferroptosis susceptibility,^[^ ^17^, ^47^, ^48^ ^]^ whereas PCBP2 regulates systemic iron trafficking by interacting with transporters such as DMT1 and ferroportin.^[^ ^48^ ^]^ This functional specialization establishes PCBP1 as the key regulator of ferroptosis sensitivity. Based on our experimental results, we believe that changes of PCBP1 expression in astrocytes are likely obscured by alterations in other cell types. Various stressors such as LPS,^[^ ^49^ ^]^ chronic alcohol,^[^ ^50^ ^]^ and high glucose exposure^[^ ^51^ ^]^ have been shown to affect PCBP1 expression. Moreover, PCBP1 is also implicated in various neurodegenerative diseases like Parkinson's,^[^ ^18^ ^]^ Alzheimer's,^[^ ^19^ ^]^ and Huntington's.^[^ ^20^ ^]^ Nevertheless, the alterations and underlying mechanisms of PCBP1 in depressive‐like behaviors induced by chronic stress remain inconclusive. Subthreshold stress paradigms are widely used in previous studies to investigate mechanisms of stress susceptibility.^[^ ^34^ ^]^ Our investigation revealed that subthreshold stress induced depressive‐like behaviors only in mice with a targeted knockdown of PCBP1 in astrocytes. Additionally, the overexpression of PCBP1 in astrocytes was observed to counteract the CUMS‐induced reduction in GPX4 levels and attenuate the depressive‐like behaviors correlated with CUMS. These results highlight the critical role of PCBP1 in averting ferroptosis within astrocytes and mitigating depressive‐like behaviors induced by CUMS.

Astrocytes maintain extracellular glutamate homeostasis via GLT1‐mediated uptake and GS‐mediated conversion to glutamine.^[^ ^33^, ^52^ ^]^ Impairment of astrocytic function hinders these processes, leading to accumulation of extracellular glutamate. This accumulation induces neuronal toxicity and compromises synaptic plasticity, thus contributing to the pathogenesis of depression.^[^ ^53^, ^54^ ^]^ Our study revealed that CUMS significantly elevated glutamate levels in vHip, primarily through impaired astrocytic clearance rather than enhanced presynaptic release. Despite reduced neuronal activity (decreased c‐Fos expression and Ca^2^⁺ signaling) and lower presynaptic release (decreased mEPSC frequency), the marked downregulation of both GLT1 and GS compromised glutamate clearance at both uptake and metabolic levels. Furthermore, prolonged decay tau of glutamate signaling demonstrated impaired clearance kinetics, establishing astrocyte dysfunction as the primary driver of glutamate accumulation. Our investigation suggested ferroptosis as a critical contributor to astrocytic glutamate metabolic dysfunction. In vitro co‐culture experiments demonstrated that erastin‐induced ferroptosis in GL261 cells abolished their neuroprotective capacity against glutamate excitotoxicity through GS activity suppression, an effect that was reversed by DFO treatment. In vivo experiments demonstrated that CUMS triggered astrocytic ferroptosis in vHip, accompanied by downregulated GLT1 and GS expression and elevated glutamate levels. Notably, both DFO administration and astrocyte‐specific PCBP1 overexpression ameliorated ferroptosis markers, restored GLT1/GS expression and glutamate homeostasis. This temporal and spatial coordination between ferroptosis inhibition and glutamate metabolism restoration establishes a closed mechanistic link between astrocytic ferroptosis and impaired glutamate clearance, rather than representing independent consequences of chronic stress. Mechanistically, ferroptosis likely disrupts glutamate homeostasis through lipid peroxidation‐mediated impairment of GLT1 localization and function,^[^ ^55^ ^]^ lipid peroxidation products‐induced suppression of GS/GLT1 expression and activity,^[^ ^56^, ^57^, ^58^, ^59^ ^]^ and astrocytic process retraction that reduces surface area available for glutamate uptake. Impaired glutamate clearance caused by astrocytic ferroptosis further affected neuronal structure and function. CUMS preferentially impaired glutamatergic neurons rather than GABAergic neurons, consistent with the known heightened susceptibility of excitatory neurons to glutamate excitotoxicity due to their abundant expression of ionotropic glutamate receptors and dependence on astrocytic glutamate uptake.^[^ ^60^, ^61^ ^]^ This demonstrated that glutamate accumulation likely contributed to the neuronal damage, as opposed to nonspecific widespread injury to neurons or glia from chronic stress.^[^ ^62^, ^63^ ^]^ Furthermore, DFO intervention or astrocyte‐specific PCBP1 overexpression improved neuronal structural and functional abnormalities and depressive‐like behaviors by inhibiting ferroptosis and restoring glutamate homeostasis. These multi‐level improvements provide strong evidence for a causal relationship between astrocytic ferroptosis, impaired glutamate clearance, and synaptic dysfunction.

While our research confirms that PCBP1 plays a role in mediating stress‐induced astrocytic ferroptosis, the specific mechanisms underlying this process remain to be fully elucidated. PCBP1 regulates mRNA stabilization and translational activation by binding polycytosine (poly‐C) with high affinity and sequence specificity.^[^ ^17^ ^]^ To investigate the underlying mechanisms, RNA sequencing was performed in astrocytes (GL261 cells) with PCBP1 knockdown. Our findings demonstrated that PCBP1 knockdown led to decreased expression of critical genes involved in iron homeostasis, such as ferritin, divalent metal transporter 1 (DMT1), and LCN2. Ferritin (Fth and Ftl subunits) stores iron in a non‐toxic form, DMT1 (also known as Slc11a2) mediates iron uptake, and LCN2 regulates iron trafficking. Disruption of these proteins impairs iron storage and transport, elevating cytoplasmic iron and ROS levels, which in turn promote lipid peroxidation and DNA damage.^[^ ^64^, ^65^, ^66^, ^67^ ^]^ Therefore, we speculate that PCBP1 downregulation disrupts iron homeostasis by reducing the expression of iron‐regulatory proteins, thereby increasing cellular susceptibility to ferroptosis. In addition, ferroptosis is closely linked to impaired antioxidant defenses, particularly the system Xc^−^‐GSH‐GPX4 axis.^[^ ^68^ ^]^ PCBP1 knockdown reduced the expression of SLC7A11 and GPX4, which are key components of this system, indicating a potential impairment in the antioxidant capacity. Furthermore, PCBP1 knockdown decreased the expression of antioxidant‐related genes, including Hmox1, Atf4 and Sesn2, which are crucial in mitigating oxidative stress‐induced cellular damage.^[^ ^69^, ^70^, ^71^ ^]^ Collectively, our RNA sequencing results indicate that PCBP1 knockdown disrupts the expression of genes involved in iron metabolism and antioxidant defense, thereby sensitizing astrocytes to ferroptosis. Moreover, our RIP‐qPCR results confirmed that PCBP1 binds to mRNAs of ferroptosis‐related genes, with Ftl as a key target regulated by PCBP1. Further functional validation showed that Fth or Ftl knockdown increased ferroptosis sensitivity, whereas Slc11a2 silencing had no effect, indicating that ferritin‐mediated iron storage is more critical than iron import in PCBP1‐regulated ferroptosis. Given the bidirectional regulation of Ftl by PCBP1, these results identified Ftl as the key downstream target, while the effect of Fth likely reflects disrupted ferritin assembly and impaired iron storage rather than direct regulation by PCBP1.^[^ ^68^ ^]^ Collectively, these findings establish Ftl as the primary target through which PCBP1 regulates ferroptosis sensitivity.

The study has several limitations to consider when interpreting the results. First, we included only male mice in our experiments, while there are physiological differences between males and females, with sex hormones (such as testosterone and estrogen) influencing stress responses and drug metabolism. These disparities may lead to sex‐based variations in experimental outcomes. Second, the proteomic and phosphoproteomic analyses have elucidated a number of additional pathways involved in chronic stress, such as apoptosis, mTOR signaling, cAMP signaling, and proteins implicated in Parkinson's disease. Although these signaling pathways extend beyond the scope of this study, they are crucial for understanding the mechanisms of stress‐related depression and warrant further investigation. Finally, our experiments were performed using immortalized GL261, C8‐D1A astrocytic and HT22 neuronal cell lines, which may differ from primary hippocampal cells in gene expression, functions, and biological mechanisms. Thus, further experiments are needed to validate these results.

## 4 Conclusion

This study clarified the function of PCBP1 in conferring antidepressant‐like effects by protecting vHip glutamatergic neurons against glutamate toxicity, achieved through the inhibition of astrocytic ferroptosis under chronic stress conditions. These findings provide significant insights into the mechanisms underlying depressive symptoms and highlight astrocytic PCBP1 as a promising molecular target for treating chronic stress‐induced depression.

## 5 Experimental Section

### Animals

Male C57BL/6J mice (8 weeks old, 25±2 g), purchased from the Vital River laboratory (Beijing, China), were housed under standard environmental conditions (22 ± 2 °C, 50% ± 5% humidity, and a 12/12 h light‐dark cycle). Mice were kept in groups of four per cage in Plexiglas cages with ad libitum access to food and water prior to the experiment. Body weight and sucrose preference were measured during the first week to establish baseline values. Subsequent changes in body weight and sucrose preference were recorded weekly or biweekly. The experiments were conducted in accordance with the NIH Guide for the Care and Use of Laboratory Animals. The experiments were approved by the Biomedical Ethics Committee of Health Science Center of Xi'an Jiaotong University (approval No. XJTUAE2023‐351).

### CUMS and Subthreshold CUMS (sCUMS) Protocol

The CUMS paradigm was carried out according to the previously established protocol with minor modifications.^[^ ^72^ ^]^ Mice in the CUMS groups were exposed to two randomly selected stressors per day over 8 weeks. The stressors included water and food deprivation for 12 h, shaking for 15 min, cage tilting for 12 h, tail nipping (1 cm from the tip of the tail) for 5 min, 45 °C heat stress for 10 min, cold swimming for 5 min (at 4 °C), inversion of the light/dark cycle for 24 h, overnight illumination for 12 h, physical restraint for 4 h, noise exposure for 4 h and damp cage (200 mL water in sawdust bedding). To ensure unpredictability, the stress schedule followed a pseudorandom sequence revised weekly.

As previously reported,^[^ ^34^, ^73^, ^74^ ^]^ a modified sCUMS protocol based on the CUMS paradigm was adapted to evaluate stress susceptibility. In this study, the stressor intensity, frequency, and duration were reduced by ≈50%. The stressors included water and food deprivation for 6 h, shaking for 7 min, cage tilting for 6 h, tail nipping for 2 min, 45 °C heat stress for 5 min, 4 °C cold swimming for 2 min, illumination for 6 h, restraint for 2 h, and noise exposure for 2 h. Mice in the sCUMS group were exposed to one randomly selected stressor per day for 4 weeks.

### Drugs Preparation and Administration

The ferroptosis inhibitor deferoxamine mesylate (DFO) (HY‐B0988, MedChemExpress) and the GS inhibitor methionine sulfoximine (MSO) (HY‐W33814, MedChemExpress) were dissolved in saline. During the CUMS procedure, mice received DFO (10 mg kg^−1^, i.p.) every other day, following a dosing regimen moderately adjusted from previous studies to ensure both efficacy and tolerability.^[^ ^75^, ^76^ ^]^ MSO (10 mm, 0.2 µL per side) was infused into vHip via implanted guide cannulas 3 h prior to the behavioral tests.^[^ ^77^ ^]^

### Sucrose Preference Test (SPT)

SPT was used to assess the loss of interest in rewarding stimuli. Mice were first habituated to a 1% sucrose solution by providing two bottles of sucrose for 24 h, followed by one bottle of sucrose and one of water for another 24 h. Prior to the SPT, mice were deprived of both food and water for 24 h. During the 1.5 h preference test, mice were housed in individual cages followed by free access to two identical bottles containing either 1% sucrose solution or water. The position of the two bottles was swapped after 45 min to avoid any side preference influencing the results. The preference value was calculated as follows: sucrose consumption / (sucrose consumption + water consumption). At the end of the test, all animals were returned to their home cages with normal breeding conditions.

### Open Field Test (OFT)

OFT was used to assess exploratory activity. The mice were placed into an open field box (45 × 45 × 30 cm) under dim light for 15 min. The arena was divided into a peripheral zone and a center zone (28 × 28 cm). The locomotor activity and the movement trace of mice were recorded using ANY‐maze software (Stoelting Company, Wood Dale, IL, USA). After each test, the apparatus was cleaned with 75% ethanol to abolish the odor of the previously tested mice.

### Tail Suspension Test (TST)

TST was used to assess the despair‐like behavior. The mice were suspended by the tail from a horizontal bar (35 cm above the floor) using an adhesive tape applied 1 cm from the tip of the tail. The test session lasted for 6 min. The immobile latency for the first 2 min and the time spent immobile during the last 4 min were determined by the observer who was blind to allocations of the mice group.

### Forced Swim Test (FST)

FST was used to assess the despair‐like behavior. The mice were individually placed in a glass cylinder (diameter 15 cm, height 25 cm) filled with 15 cm of water at 24 ± 1 °C and forced to swim for 6 min. The water was changed between each trial to prevent odor contamination. The immobile latency for the first 2 min and the time spent immobile during the last 4 min were determined by the observer who was blind to allocations of the mice group. The immobility was defined as floating or only making slight movements to keep the head above the water.

To minimize observer bias during the behavioral assessments, all experiments were conducted under strict double‐blind conditions. Specifically, both the experimenters performing the tests and those analyzing the data were blinded to group allocations.

### Quantitative Proteomics and Phosphoproteomics

The tissue samples of vHip were collected from three mice per group and pooled as one sample. The samples were preprocessed through protein extraction, protein digestion, TMT labeling, enrichment of modified peptides and fraction separation. Liquid chromatography‐tandem mass spectrometry (LC‐MS/MS) was performed using a Q Exactive Plus mass spectrometer coupled with an Easy 1200 nLC (Thermo Fisher Scientific) in Beijing Novogene Biotechnology Co., Ltd (China).

### Sequence Database Searching

To identify proteins and peptides, the Proteome Discoverer version 2.4 software was used to analyze the raw mass spectrum (MS) data. Trypsin was selected as the digestion enzyme, and the MS spectra were searched against the Mus musculus Uniprot FASTA database (release 2020_7_2, containing 86555 sequences). A maximum of two missed cleavages were allowed, with the following search parameters: precursor ion mass tolerance of 10 ppm and product ion mass tolerance of 0.02 Da.

For proteomic analysis, carbamidomethylation was set as a fixed modification, while oxidation of methionine (M) and TMT plex were set as dynamic modifications. Acetylation, TMT plex, Met‐loss and Met‐loss+Acetyl were specified as N‐Terminal modifications. For phosphoproteomic analysis, carbamidomethylation was specified as fixed modifications, with oxidation of methionine (M), phosphorylation of serine (S), threonine (T), and tyrosine (Y), as well as N‐terminus acetylation were specified as variable modifications. Proteome Discoverer version 2.4 further filtered and optimized the analysis results: Peptide Spectrum Matches (PSMs) with a confidence level greater than 99% were considered identified. The identified protein contains at least 1 unique peptide. Only identified PSMs and proteins with a false discovery rate (FDR) of less than 1.0% were retained for further analysis.

### Data Analysis

For proteomics, a total of 7190 proteins were identified. The relative quantification of proteins was statistically analyzed using a Student's t‐test, based on the average of normalized protein abundance values. *P* < 0.05 was considered statistically significant. The proteins with a 1.1‐fold change (FC) and *P* < 0.05 were classified as the statistically significant differentially expressed proteins (DEPs). The upregulated proteins were identified as FC > 1.1 and the downregulated proteins were identified as FC < 0.9.

For phosphoproteomics, a total of 2012 phosphoproteins and 5143 phosphorylated modification sites were identified in the fractions at 1% FDR. Student's t‐test was then performed to compare the two groups, using a *P* value of < 0.05 and a fold‐change threshold of 1.2 for upregulation or 0.83 for downregulation as criteria for significance. Proteins with a FC > 1.2 were considered upregulated, while those with a FC < 0.83 were considered downregulated.

### Bioinformatics Analysis

Annotated via UniProtKB/Swiss‐Prot, Gene Ontology (GO) and Kyoto Encyclopedia of Genes and Genomes (KEGG), the proteins were subjected to enrichment analysis. The enriched pathways in GO biological processes and KEGG were identified and listed according to their enrichment *P*‐value (*P* < 0.05). The mass spectrometry proteomics data had been deposited to the ProteomeXchange Consortium (http://proteomecentral.proteomexchange.org) via the iProX partner repository, with the dataset identifier PXD059700.

### Immunostaining Analysis

After the final behavioral test, mice were intracardially perfused with 0.01 m phosphate‐buffered saline (PBS) and 4% paraformaldehyde (PFA) in PBS. For frozen sections, the brains were post‐fixed overnight in 4% PFA and then dehydrated in 30% sucrose at 4 °C. The entire brain was serially sectioned into 30‐µm‐thick transverse slices using a freezing microtome (Leica, Germany). For paraffin sections, the brains were embedded in paraffin (Thermo, USA), and then serially sectioned into 5‐µm‐thick transverse slices.

For immunofluorescence analysis, the 30‐µm‐thick sections were incubated overnight at 4 °C with primary antibodies against NEUN (94403S, Cell Signaling Technology), GFAP (GB11096, Servicebio), IBA‐1 (GB12105, Servicebio), CaMKII (11533‐1‐AP, Proteintech), GAD67 (PA5‐21397, Invitrogen), c‐Fos (OB‐PGP080, Oasis biogarm), GPX4 (ab125066, Abcam), and PCBP1 (14523‐1‐AP, Proteintech). The sections were then incubated with fluorescence‐conjugated secondary antibodies at room temperature for 4 h. All sections were stained with DAPI for 10 min. For cell death detection, the 5‐µm‐thick sections were stained using the TUNEL kit (A112, Vazyme). The slides were observed under the fluorescence microscope (Zeiss, Germany) and analyzed using Image J software. The morphometric characteristics of GFAP‐positive cells were analyzed using the Sholl and skeleton analysis functions in ImageJ software.

### Western Blot

At the end of the behavioral test, the mice were killed and the vHip tissue was quickly harvested and placed on dry ice. The tissue was stored at −80 °C until further analysis. Western blot was performed as previously described.^[^ ^78^ ^]^ The primary antibodies used were as follows: anti‐GRIA1 (A1826, Abclonal), anti‐GRIA3 (A13993, Abclonal), anti‐DHCR7 (ab103296, Abcam), anti‐GS (GB121177, Servicebio), anti‐14‐3‐3γ (A21651, Abclonal), anti‐GPX4 (ab125066, Abcam), anti‐PCBP1 (14523‐1‐AP, Proteintech), anti‐PCBP2 (CY7300, Abways), anti‐PSD95 (A0131, Abclonal), anti‐SYN (GB15814, Servicebio), anti‐GLT1 (CY6868, Abways), anti‐GLAST (CY6874, Abways), anti‐Fth (CY7085, Abways), anti‐Ftl (CY6955, Abways), anti‐Slc11a2 (20507‐1‐AP, Proteintech), and anti‐GAPDH (GB15002, Servicebio). Phosphorylated proteins were examined by Phos‐tag SDS‐PAGE (PA101, Vazyme). Signals were developed using the enhanced chemiluminescence kit (Millipore, Billerica, USA). Densitometry analysis was performed to quantify signal intensity using Image Lab software (Bio‐Rad, USA).

### Transmission Electron Microscope (TEM)

Mice were intracardially perfused with a mixture of 2.5% glutaraldehyde and 2.5% PFA. The bilateral vHip was then carefully removed and post‐fixed with a mixture of 4% PFA and 0.25% glutaraldehyde. The samples were fixed in a fresh solution of 1% osmium tetroxide for 90 min, followed by dehydration in ethanol and embedding with low viscosity resin. Ultrathin sections were prepared, collected on a 200‐mesh grid, and stained with 2% uranyl acetate and lead citrate for 30 min. Sections were photographed under the JEOL JEM‐100SX transmission electron microscope (Hitachi, Ltd., Tokyo, Japan). Changes in synaptic structures of neurons were quantitatively analyzed, while the mitochondrial morphology of astrocytes was qualitatively described. Astrocytes were identified by the presence of cytoplasmic glial filaments, a well‐established ultrastructural marker.

### Golgi‐Cox Staining

The Golgi‐Cox staining was performed following the protocol described by Zaqout's study.^[^ ^79^ ^]^ After transcardial perfusion with 0.9% saline, vHip tissues were collected and immersed in Golgi‐Cox solution in the dark for 10 d. The tissues were then stored in a tissue‐protectant solution at 4 °C in the dark for 7 d. The 100‐µm coronal sections containing the vHip were collected using the vibratome. The sections were stained with a 3:1 ammonia solution and 5% sodium thiosulfate first. Then dehydrated in ethanol, cleared in xylene solution and sealed with neutral resin. Z‐stacks images of Golgi‐stained sections were captured using a fluorescence microscope (Zeiss, Germany) and analyzed with Image J software.

### Glutamate, Malondialdehyde (MDA), Glutathione (GSH) and Iron Assay

The vHip tissues were homogenized and centrifuged at 12000 ×*g* for 15 min at 4 °C. The levels of glutamate, MDA, GSH and iron were measured using corresponding commercially available kits (Nanjing Jiancheng Bioengineering Institute, China). All procedures were performed following the manufacturer's instructions.

### Reactive Oxygen Species (ROS) Assay

The levels of reactive oxygen species (ROS) were detected using Dihydroethidium (D7008, Sigma) for brain sections and H2DCFDA (HY‐D0940, MedChemExpress) for cells. After two washes with PBS, the brain sections and cells cultured on coverslips were incubated with Dihydroethidium or H2DCFDA for 30 min and stained with DAPI (DY20101, DIYIBIO) for 10 min at 37 °C in the dark. The fluorescence images of ROS were captured using a fluorescence microscope (Zeiss, Germany) and the mean fluorescence intensity was analyzed with Image J software.

### Stereotaxic Surgery and Virus Injection

The virus AAV2/5‐GfaABC1D‐shPCBP1‐eGFP and AAV2/9‐CaMKII‐eGFP were purchased from OBio Technology (Shanghai, China). The viruses AAV2/9‐GFAP‐PCBP1‐mCherry, AAV2/9‐CaMKII‐GCaMP6s, and AAV2/9‐CaMKII‐eGFP were purchased from HanBio (Shanghai, China). The virus AAV2/9‐hSyn‐iGluSnFR and AAV2/9‐hSyn‐eGFP were purchased from Brain Case (Shenzhen, China). For viral injection, mice were anesthetized with 2% isoflurane and placed in a stereotaxic device (RWD, Shenzhen, China). AAV (300 nL per side) was bilaterally injected into the vHip (AP –3.5 mm and ML ±3.3 mm) at three depths (DV −4.4, −3.7, and −3.1 mm), with 100 nL delivered at each site over 10 min. After the infusion, the microsyringe was left in place for an additional 5 min to facilitate virus diffusion. On day 49 of the CUMS procedure, bilateral guide cannulas (RWD, Shenzhen, China) were stereotaxically implanted into the vHip (AP ‐3.5 mm, ML ±3.3 mm, DV −3.7 mm) under anesthesia and secured with dental cement for subsequent infusion experiments.

### Fiber Photometry Recording

After 4 weeks of stress, mice injected with AAV2/9‐GFAP‐PCBP1‐mCherry or control vector were unilaterally injected with 300 nL of AAV2/9‐CaMKII‐GCaMP6s, AAV2/9‐hSyn‐iGluSnFR or control vector into the vHip, then the optical fiber (400 µm core, 0.5 NA, RWD) was implanted and secured using dental cement. Fluorescence signals (GCaMP6s or iGluSnFR) were recorded using a fiber photometry system (R810, RWD Life Science, China), which utilized two continuous sinusoidally modulated LEDs at 470 nm (calcium/glutamate‐dependent) and 410 nm (isosbestic control, calcium/glutamate‐independent) to excite GCaMP6s or iGluSnFR. The 410 nm signal was used to correct for motion artifacts, photobleaching, and autofluorescence. After 8 weeks of the CUMS procedure, mice were connected to the fiber patch cord and allowed to habituate for 5 min prior to each behavioral testing session. Then, the mice were subjected to TST or FST for six min and the fiber photometry data were recorded continuously during the tests. The resulting fitted 410 nm signal was then used to normalize the 470 nm signal as follows: x = ΔF/F = (470 nm signal – fitted 410 nm signal) / fitted 410 nm signal. The standard Z score was calculated as follows: Z score = (x‐mean) / std, x = ΔF/F. Glutamate transient events were detected using an automated threshold‐based algorithm (baseline mean + 3SD, ΔF/F ≥ 2). For each detected event, the event frequency (events/min), area under the curve (AUC), and decay time constant (tau, single exponential fit) were quantified. All analyses were performed using custom scripts in R software (version 4.3.3).

### Cell Culture and Transfection, and Viral Infection

GL261 murine astrocytic cell lines (RRID: CVCL_Y003, purchased from Fenghbio, Hunan, China), C8‐D1A murine astrocytic cell lines (RRID: CVCL_6379, purchased from Procell, Hubei, China) and murine neuronal HT‐22 cell lines (RRID: CVCL_0321, a kind gift from Dr. Bao Zhang, Xi'an Jiaotong University) were cultured in DMEM (Gibco) supplemented with 10% fetal bovine serum (FBS) (Gibco) and 1% Penicillin‐Streptomycin Solution (HyClone, SV30010). The cells were maintained in an incubator at 37 °C with 5% CO_2_.

Cell transfections were performed using Lipofectamine TM 2000 (Invitrogen, 11 668 027) according to the manufacturer's instructions. The PCBP1 shRNA sequence (shPCBP1: 5′‐CCATGAACTCACCATTCCAAA‐3′), PCBP2 shRNA sequence (shPCBP2: 5′‐GCAGCTCTATGACCAATAGTA‐3′), Fth siRNA sequences (sense 5′‐GAAUCAGUCACUACUGGAA dTdT‐3′ and antisense 5′ ‐UUCCAGUAGUGACUGAUUC dTdT‐3′), Ftl siRNA sequences (sense 5′ ‐GAGGUGAAACUCAUCAAGA dTdT‐3′ and antisense 5′ ‐UCUUGAUGAGUUUCACCUC dTdT‐3′) and Slc11a2 siRNA sequences (sense 5′ ‐CCUGAGGAGGAGUACUCUU dTdT‐3′ and antisense 5′‐AAGAGUACUCCUCCUCAGG dTdT‐3′) were synthesized by Tsingke (Xi'an, China). For cell viral infection, the PCBP1‐OE lentivirus was synthesized by Hanbio (Shanghai, China). The GL261 cells and C8‐D1A cells were infected with PCBP1‐OE lentivirus using polybrene reagent. Stably expressing clones were selected using puromycin dihydrochloride (ST551, Beyotime, China).

### Cell Viability Assay

Following previous studies with minor modifications, cells were treated with erastin^[^ ^80^ ^]^ (HY‐15763, MedChemExpress) (5–15 µm for GL261 cells, 10 µm for C8‐D1A cells) or 20–25 mm glutamate^[^ ^31^ ^]^ (IG0710, Solarbio) for HT22 cells, followed by cell viability assessment using the Cell Counting Kit‐8 (G4103, Servicebio, China) according to the manufacturer's instructions. Absorbance values at 450 nm were acquired using an enzyme‐labeled instrument. Each experimental group included three biological replicates.

### Activity of Glutamine Synthetase and Lactate Dehydrogenase Assay

GL261 or HT22 cells were seeded into plates. After the interventions of erastin or glutamate, the activities of glutamine synthetase (GS) and lactate dehydrogenase (LDH) were measured using the GS activity assay kit (BC0910, Solarbio) and LDH assay kit (BC0685, Solarbio) according to the manufacturer's instructions. Each experimental group included three biological replicates.

### Co‐Culture Experiments

The cell co‐culture experiment was conducted following the method described by Allaman.^[^ ^81^ ^]^ In brief, HT22 cells were plated in the culture dishes and GL261 cells were seeded onto the coverslips at a ratio of 2:1 (GL261 to HT22 cells). Three sterile paraffin dots were added to each dish before seeding to prevent direct contact between the two cell types. After 24 h of seeding, the co‐culture system was established by carefully transferring GL261 cell‐covered coverslips on top of the HT22 cells layer cultured in the dish. The glutamate was added to the co‐culture system. After 24 h of co‐culture, the GL261 cell‐covered coverslips were removed and the cell viability, lactate dehydrogenase activity and protein expression levels of the HT22 cells were assessed.

### Whole‐Cell Patch Clamp Recording

Mice were anesthetized with isoflurane and intracardially perfused with cold artificial cerebrospinal fluid (ACSF). Brain slices (300 µm) containing vHip were sectioned using the vibratome VT1200S (Leica, Wetzlar, Germany). After recovery for 45 min at room temperature in ACSF, the brain slices were transferred to the recording chamber with continuous ACSF perfusion. eGFP‐expressing neurons were visualized using the fluorescence microscope (Nikon, Japan). The membrane potential was held at −70 mV. Action potentials were recorded under control conditions, and miniature excitatory postsynaptic currents (mEPSC) were recorded in the presence of tetrodotoxin (TTX, 1 µM).

### Transcriptome Analysis

Transcriptome sequencing of RNA extracted from GL261 cells was performed by Novogene (Beijing, China; https://cn.novogene.com). RNA integrity was assessed using an Agilent 2100 bioanalyzer. The sequencing libraries were generated following mRNA enrichment. After quality control, the different libraries were pooled and sequenced on an Illumina platform based on the principle of “Sequencing by Synthesis”. After pre‐processing by removing reads containing adapters, reads containing poly‐N and low‐quality reads, high‐quality data in FASTQ format were obtained. The raw transcriptome reads were mapped to the reference gene sequences using Hisat2 v2.0.5, and the number of reads mapped to each gene was quantified using featureCounts v1.5.0‐p3. Gene expression levels were calculated as FPKM based on the gene length and the number of mapped reads. Differential expression analysis between the two groups was conducted using the DESeq2 R package (v2.12), with a corrected *P*‐value < 0.01 set as the threshold for significant differential gene expression. The enriched pathways of GO and KEGG were identified and listed using the clusterProfiler R package (v3.8.1) and ranked by enrichment significance (*P* < 0.05). Raw transcriptomic data had been deposited in the NCBI Sequence Read Archive (SRA) under the accession number PRJNA1209353.

### Motif Analysis

Motif discovery was performed using the MEME software. The 1‐kb sequence upstream of the transcription start site (TSS) was downloaded from the Genome Reference Consortium Mouse Build 39. MEME was utilized to identify the top 10 enriched motifs among these differentially expressed genes, representing potential regulatory elements associated with PCBP1. Subsequently, to further assess motif similarity, the TomTom tool was employed to compare the discovered motifs with known PCBP1 binding motifs.

### RNA Isolation, Reverse Transcription and qPCR

Total RNA was extracted from cell samples using TRIzol reagent. The purified RNA was then reverse transcribed into cDNA using the *Evo M‐MLV* RT Mix kit (AG11728, Agbio) following the manufacturer's instructions. Quantitative PCR was performed using SYBR Green qPCR Master Mix (AG11718, Agbio), with GAPDH serving as the internal reference gene. Primer sequences are listed in Table S5 (Supporting Information).

### RNA Immunoprecipitation (RIP) Assay

RNA immunoprecipitation was performed using the RIP Kit (Bersin Biotechnology, China) according to the manufacturer's instructions. GL261 cells were lysed in RIP lysis buffer and incubated with magnetic beads conjugated with either anti‐PCBP1 antibody or normal IgG (negative control) at 4 °C. After washing, RNA‐protein complexes were eluted, and RNA was extracted using TRIzol reagent. Enriched RNAs were reverse transcribed and analyzed by RT‐qPCR.

### PCR Amplification

PCR was performed using gene‐specific primers under standard cycling conditions. Amplified products were visualized on 2% agarose gels and visualized by ethidium bromide staining.

### Statistical Analysis

Data analysis and graphical visualization were performed using GraphPad Prism 8.0 and R software. Prior to statistical testing, normality and homogeneity of variance were assessed via the Shapiro‐Wilk and Levene's tests. For normally distributed and homoscedastic data, unpaired t‐test (two groups) or one‐way/two‐way ANOVA (with ordinary or repeated measures) was employed, followed by Tukey's, Sidak's, or Dunnett's post hoc tests as appropriate. Welch's t‐test was used for normally distributed but heteroscedastic data. For non‐normal data, the nonparametric Mann‐Whitney U test (two groups) or Kruskal‐Wallis test (multi‐groups) was applied. All parametric tests were two‐tailed, with statistical significance set at *P* < 0.05 (*P < 0.05, **P < 0.01, ***P < 0.001, ****P < 0.0001). Sample sizes (n) for each experiment are indicated in the corresponding figure legends, representing the number of independent biological replicates. All data were presented as means ± SEM. Detailed statistical parameters are provided in the Table S6 (Supporting Information).

## Conflict of Interest

The authors declare no conflict of interest.

## Author Contributions

Y.W. performed conceptualization. J.Z., B.Z., M.J., Y.Z., Y.L., W.X., Z.Z., X.G., Q.M., Y.G., Y.G., P.L., and F.Z. performed data acquisition and investigation. Y.W. and J.Z. performed writing and supervision. Y.W., X.M., and S.W. performed reviewing and editing.

## Supporting information



Supporting Information

Supplementary Tables 1‐6

## Data Availability

The data that support the findings of this study are available from the corresponding author upon reasonable request.
