# Supplementary material for: Astrocytic PCBP1 Suppresses Ferroptosis to Restore Glutamatergic Homeostasis and Mitigate Stress‐Induced Depression in Male Mice
Source: Adv Sci (Weinh). 2025 Dec 12;13(10):e13438. doi: 10.1002/advs.202513438 (PMC12915190; doi:10.1002/advs.202513438)
Supplement: Supplementary file 1 — Supporting Information [file ADVS-13-e13438-s002.docx]

Supporting Information

**Astrocytic PCBP1 Suppresses Ferroptosis to Restore Glutamatergic Homeostasis and Mitigate Stress-Induced Depression in Male Mice**

*Jinyu Zhang, Binbin Zhao, Min Jia, Yan Zhao, Ye Lu, Wenyu Xi, Ziyu Zhu, Xiaojuan Gong, Qingyan Ma, Yuan Gao, Yijie Guo, Pan Li, Feng Zhu, Shuguang Wei^*^, Xiancang Ma^*^, Yunpeng Wang^*^*

Figure S1

Figure S2

Figure S3

Figure S4

Figure S5

Figure S6

Figure S7

Figure S8

Table S1-DEPs

Table S2-GO-KEGG

Table S3-DEGs

Table S4-GO-KEGG

Table S5-PCR primer sequence

Table S6-Statstical results


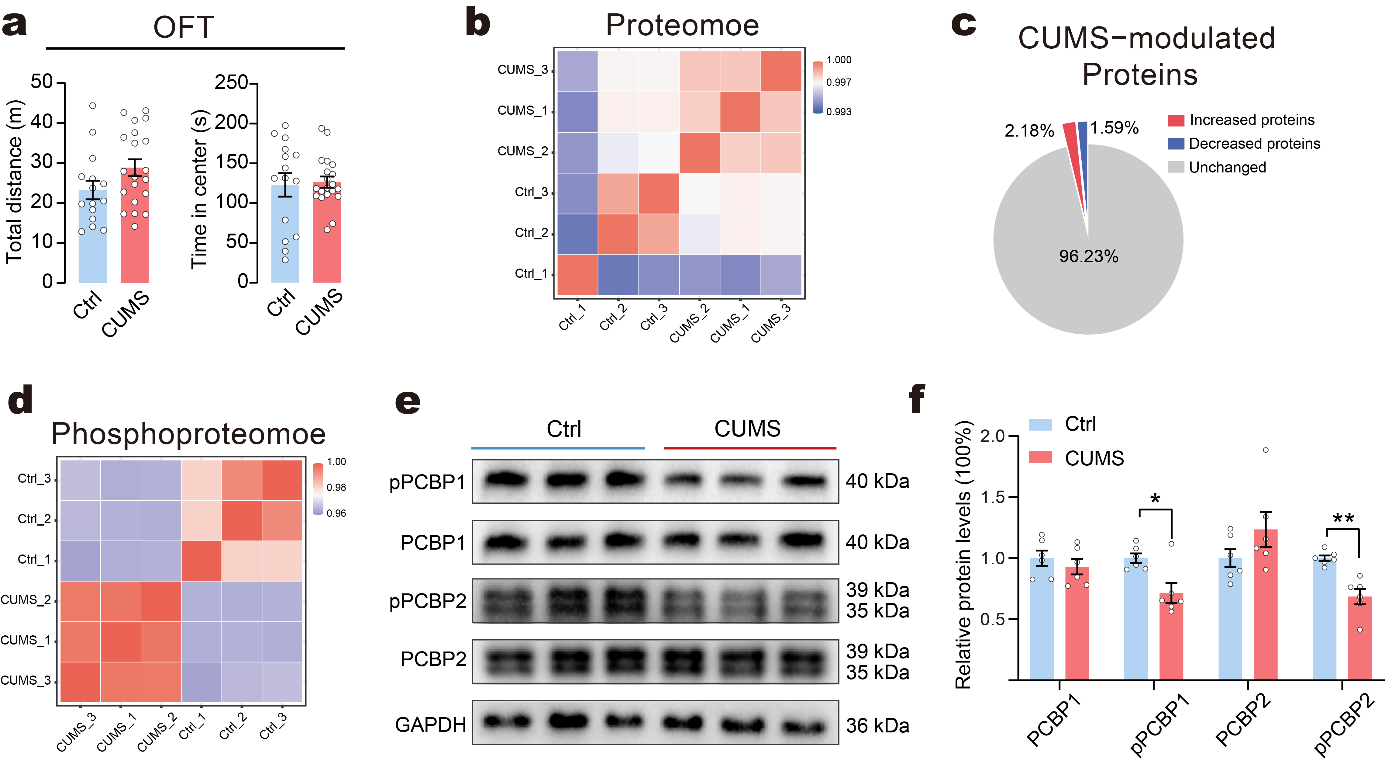


**Figure S1. Locomotor activity in mice and analysis of proteomics and phosphoproteomics.**

(a) Locomotor activity in OFT. n = 15-20 mice/group. (b) Pearson correlation analysis for proteomics. (c) Distribution of proteins modulated by CUMS. (d) Pearson correlation analysis for phosphoproteomics. (e) Representative immunoblot images for phospho-PCBP1 and phospho-PCBP2. (f) Expression levels of the phosphoproteins validated by immunoblotting. n = 6 samples/group for analysis. Statistical analyses included unpaired t-test, Welch’s test and Mann-Whitney test. Data are presented as means ± SEM. **P* < 0.05, ***P* < 0.01.


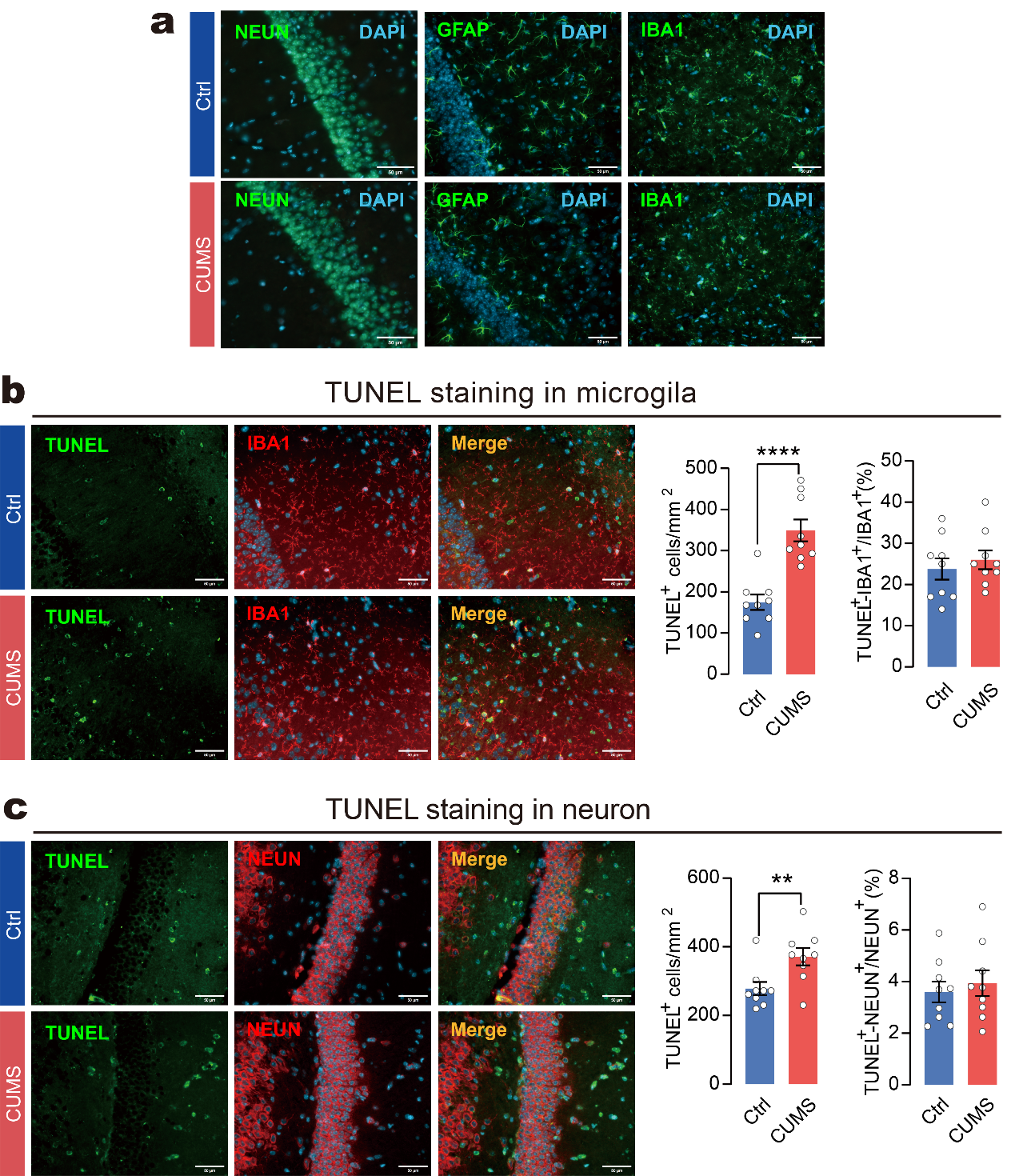


**Figure S2. Immunofluorescence images of cell-type markers and TUNEL expression in the vHip.**

(a) Representative images of neurons, astrocytes and microglia in vHip. (b-c) Measurement of apoptosis by TUNEL fluorescence staining (green) in microglia (b) and neurons (c) in vHip. Scale bar = 50 μm. n = 9 slices from 3 animals/group. Statistical analysis was performed using unpaired t-test. Data are presented as means ± SEM. ***P* < 0.01, *****P* < 0.0001.


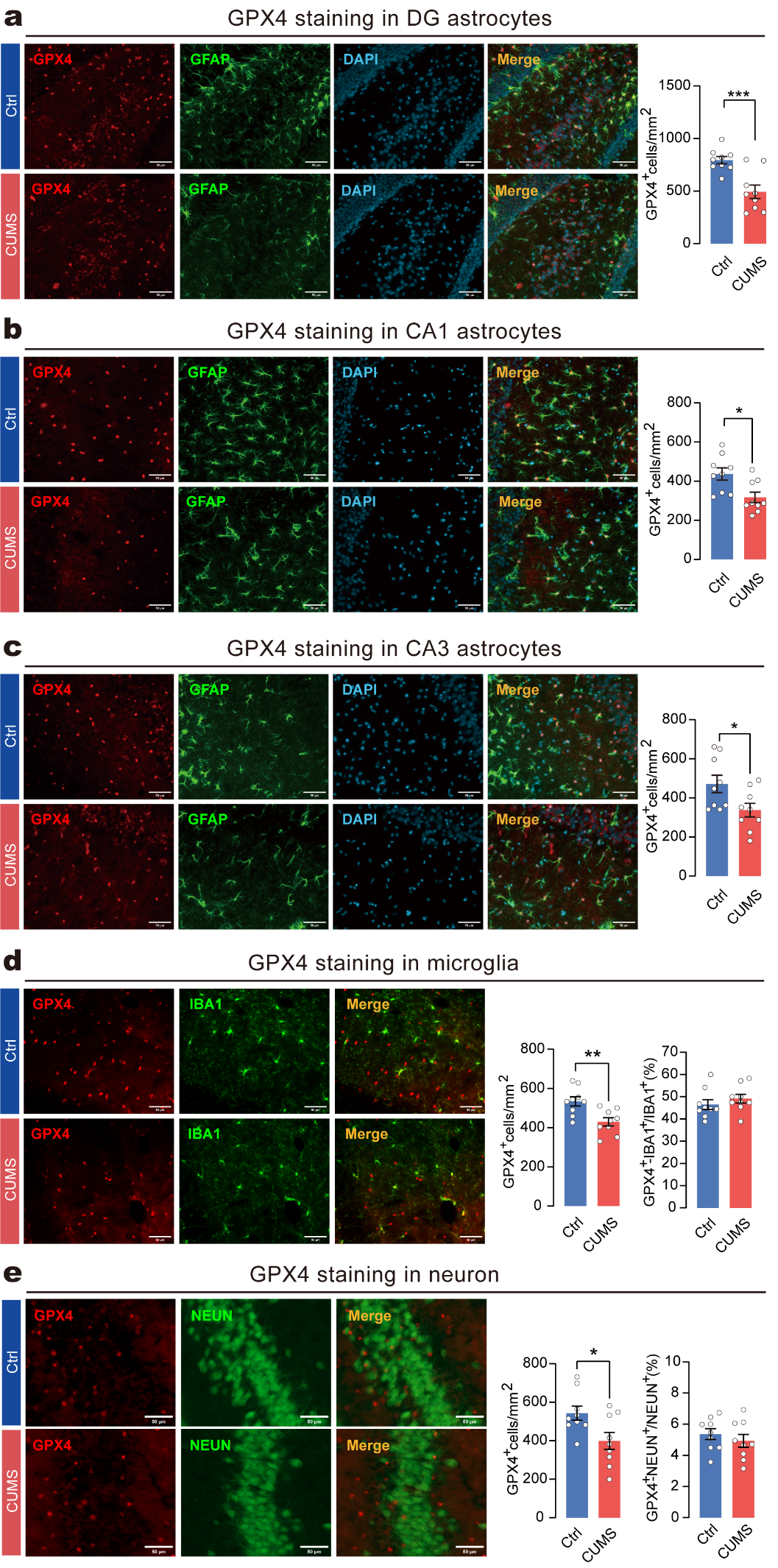


**Figure S3. Immunofluorescence images of GPX4 expression in the vHip.**

(a-c) Representative images (red, GPX4; green, GFAP) and quantification of the GPX4-positive astrocytes in DG (a), CA1 (b) and CA3(c). (d) Representative images (red, GPX4; green, IBA1) and quantification of the GPX4-positive microglia in vHip. (e) Representative images (red, GPX4; green, NEUN) and quantification of the GPX4-positive neurons in vHip. Scale bar = 50 μm. n = 9 slices from 3 animals/group. Statistical analysis was performed using unpaired t-test. Data are presented as means ± SEM. **P* < 0.05, ***P* < 0.01, ****P* < 0.001.


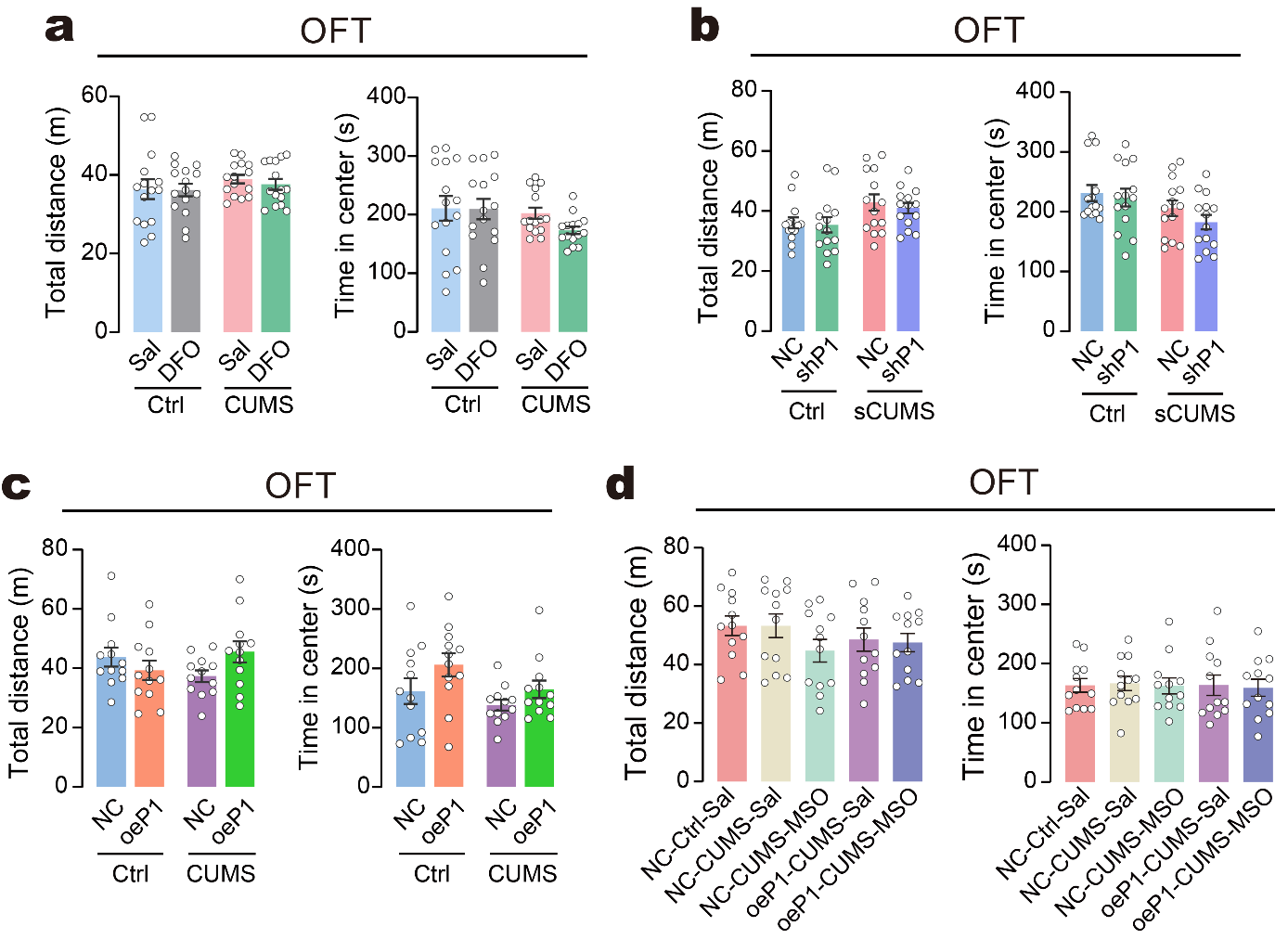


**Figure S4. Locomotor activity in mice of different experiments.**

(a) Locomotor activity in OFT of mice in DFO treatment experiments. n = 15 mice/group. (b) Locomotor activity in OFT of mice in shPCBP1 intervention experiments. n = 14 mice/group. (c) Locomotor activity in OFT of mice in oeP1 intervention experiments. n = 12 mice/group. (d) Locomotor activity in OFT of mice in MSO intervention experiments. n = 12 mice/group. Statistical analysis was performed using one-way/two-way ANOVA followed by Tukey’s post hoc test. Data are presented as means ± SEM.


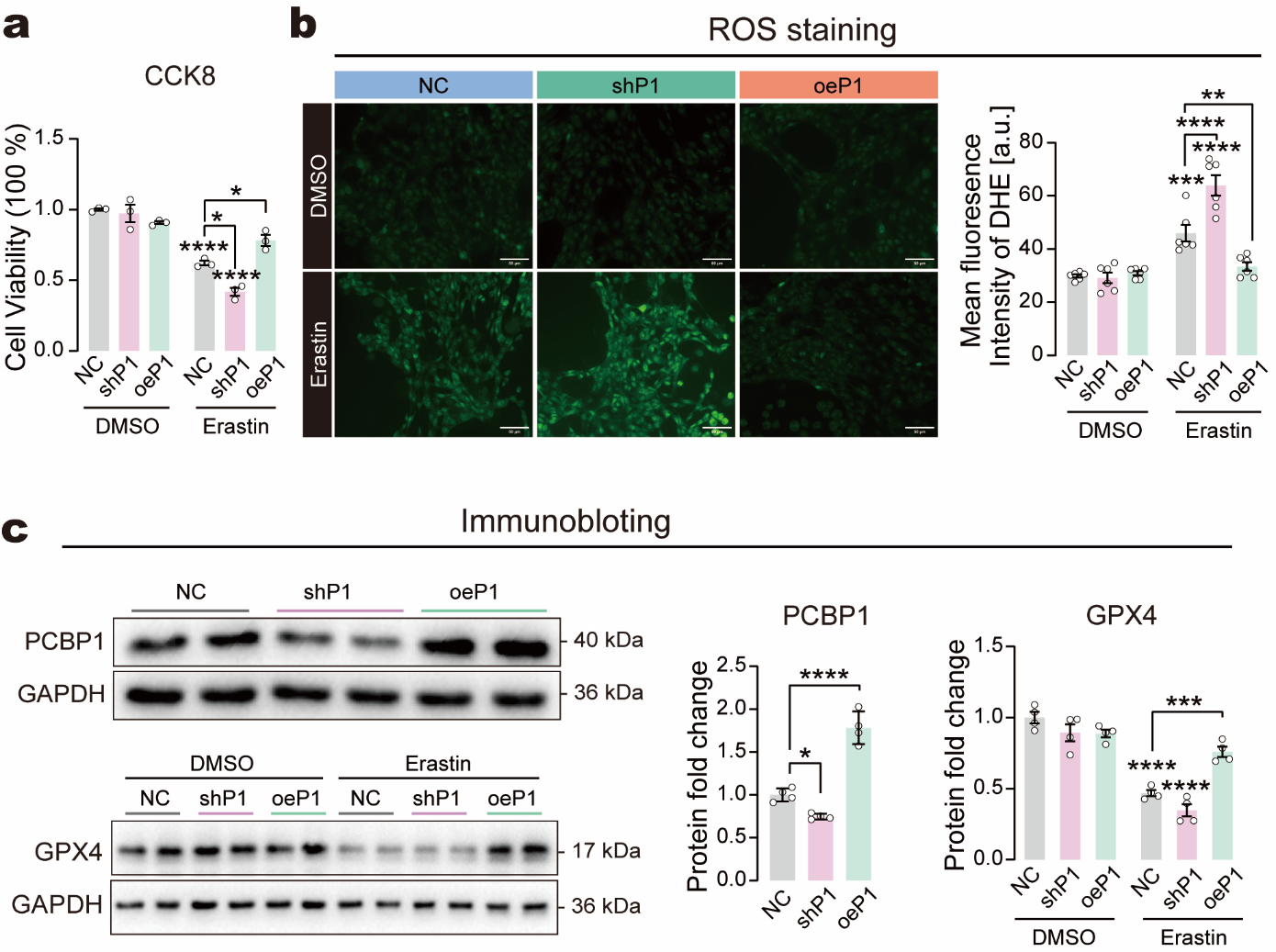


**Figure S5. PCBP1 regulates ferroptosis sensitivity in C8-D1A astrocytes.**

(a) Cell viability following erastin treatment. (b) Representative images (left) and quantification (right) of DHE fluorescence indicating ROS levels. Scale bars = 50 μm. (c) Protein expression of PCBP1 and GPX4. Left: Representative immunoblot images. Right: Quantification of protein expression. n = 3-4 samples/group. Statistical analyses included one-way ANOVA followed by Dunnett’s post hoc test and two-way ANOVA followed by Tukey’s post hoc test. Data are presented as means ± SEM. **P* < 0.05, ***P* < 0.01, ****P* < 0.001, *****P* < 0.0001.


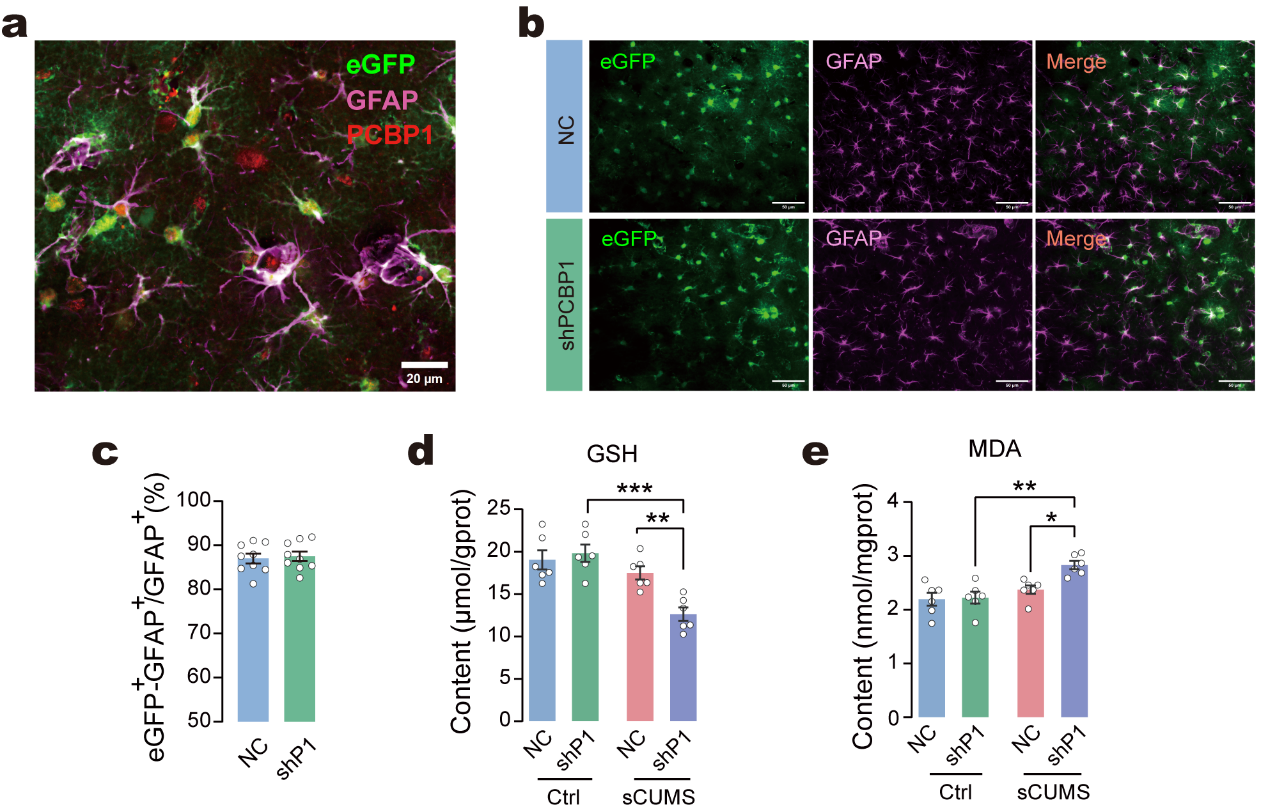


**Figure S6.** **Immunofluorescence images of eGFP expression in astrocyte and the levels of GSH and MDA in vHip.**

(a) Representative images (green, eGFP; purple, GFAP; red, PCBP1) in vHip. Scale bars = 20 μm. (b-c) Representative images (b) and quantification (c) of the eGFP-positive astrocytes. Scale bars = 50 μm. n = 9 slices from 3 animals/group. (d-e) Levels of GSH (d) and MDA (e). n = 6 samples/group. Statistical analyses included unpaired t-test and two-way ANOVA followed by Tukey’s post hoc test. Data are presented as means ± SEM. **P* < 0.05, ***P* < 0.01, ****P* < 0.001.


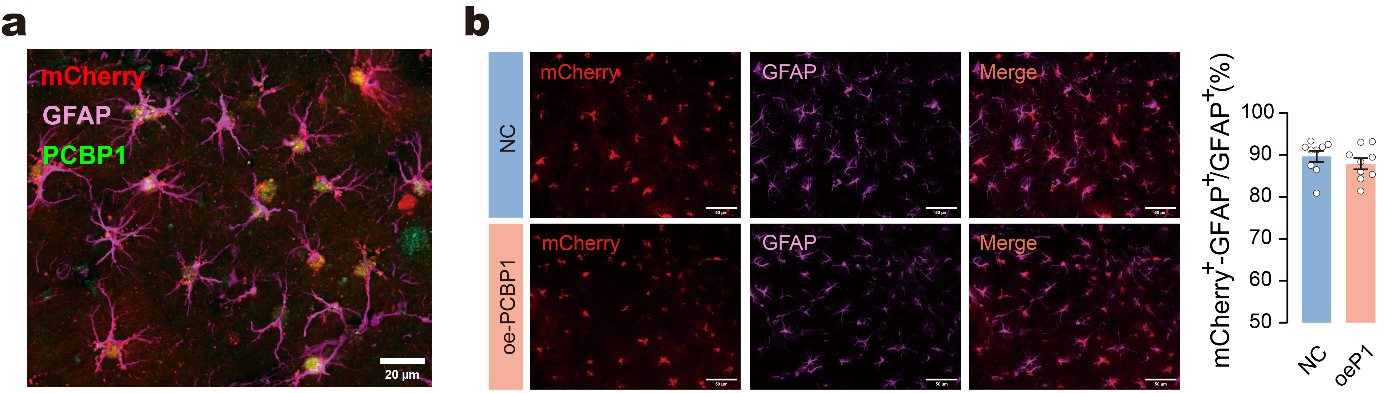


**Figure S7.** **Immunofluorescence images of mCherry expression in the vHip astrocyte.**

(a) Representative images of triple immunofluorescence co-staining (red, mCherry; purple, GFAP; green, PCBP1). Scale bars = 20 μm. (b) Representative images (left) and quantification (right) of the mCherry-positive astrocytes. Scale bars = 50 μm. n = 9 slices from 3 animals/group. Statistical analysis was performed using unpaired t-test. Data are presented as means ± SEM.


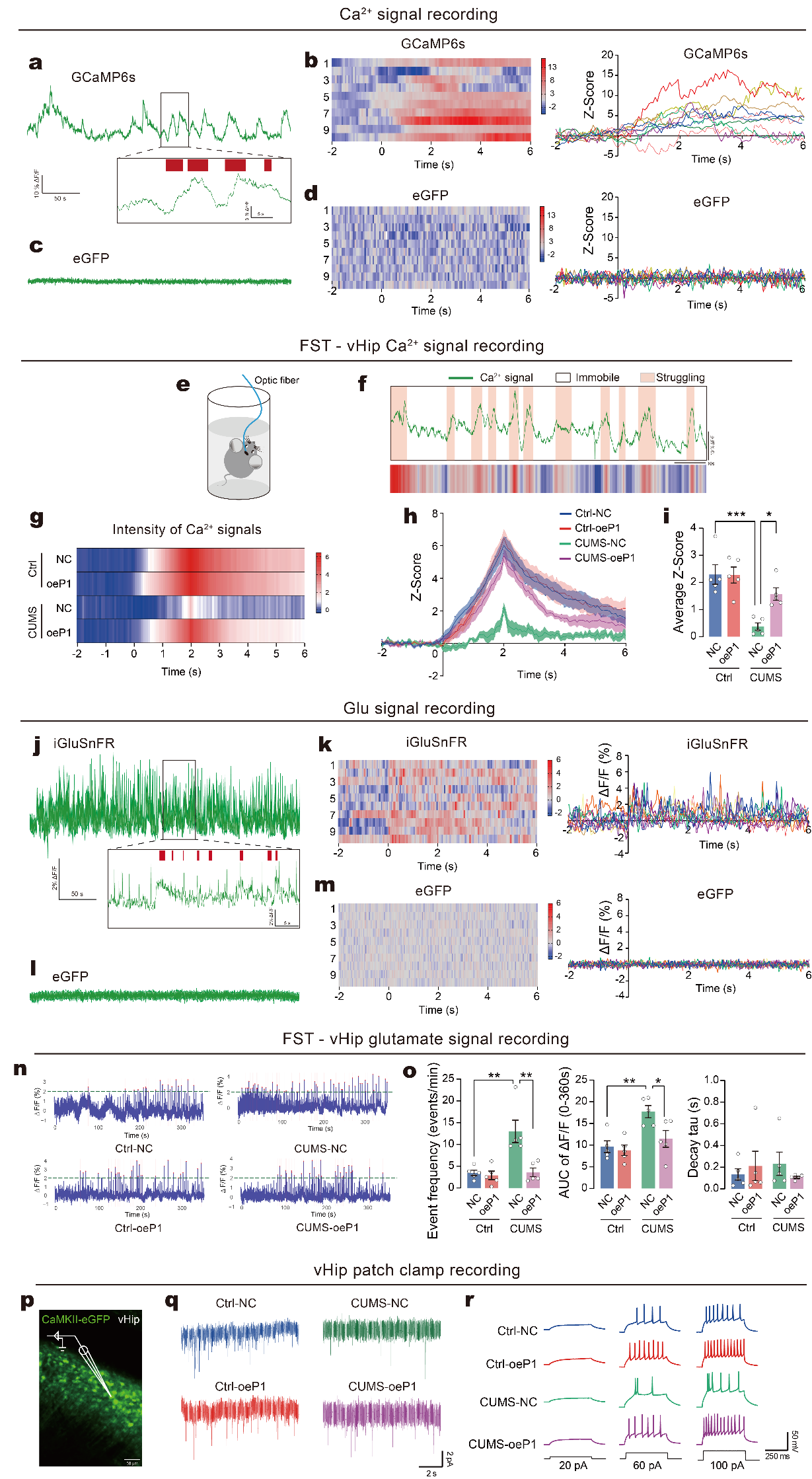


**Figure S8. Ca²⁺ and glutamate dynamics during TST or FST and electrophysiological properties of vHip glutamatergic neurons.**

(a) Representative GCaMP6s trace during TST. Red bars indicate struggling periods. (b) Example heatmap (left) and peri-event plots (right) expressing GCaMP6s aligned to struggle. (c) Representative eGFP control trace during TST. (d) Example heatmap (left) and peri-event plots (right) expressing eGFP controls aligned to struggle. (e) Schematic of fiber photometry recording in FST. (f) Representative Ca²⁺ signal traces in response to FST during the struggling and immobility phases. Scale bars = 10 s. (g-i) Ca²⁺ signal recording in vHip during FST. Representative heatmaps showing Ca²⁺ signal during struggling (g). Z-score traces and quantification of average Z-scores (h-i). n = 5 mice/group. (j) Representative iGluSnFR trace during TST. Red bars indicate struggling periods. (k) Example heatmap (left) and peri-event plots (right) expressing iGluSnFR aligned to struggle. (l) Representative eGFP control trace during TST. (m) Example heatmap (left) and peri-event plots (right) expressing eGFP controls aligned to struggle. (n) Representative trace of glutamate signal event detection during FST. Red dots indicate detected events. (o) Event-based quantification. Event frequency (left), AUC (middle) and decay tau (right). n = 5 mice/group. (p) Representative image of vHip eGFP-expressing glutamatergic neurons recorded in electrophysiological experiments. Scale bar = 50 μm. (q) Representative traces of mEPSC. (r) Representative traces of evoked action potentials. Statistical analysis was performed using two-way ANOVA followed by Tukey’s post hoc test. Data are presented as means ± SEM. **P* < 0.05, ***P* < 0.01, ****P* < 0.001.
